# CD70 recruitment to the immunological synapse is dependent on CD20 in B cells

**DOI:** 10.1073/pnas.2414002122

**Published:** 2025-04-15

**Authors:** Abbey B. Arp, Andrea Abel Gutierrez, Martin ter Beest, Guus A. Franken, Harry Warner, Andrea Rodgers Furones, Angelique N. Kenyon, Franziska Jäger, Alfredo Cabrera-Orefice, Kathrin Kläsener, Sjoerd van Deventer, Lenny Droesen, Vera Marie E. Dunlock, René Classens, Julian Staniek, Jannie Borst, Michael Reth, Ulrich Brandt, Piet Gros, Taco W. Kuijpers, Mirjam H. M. Heemskerk, Marta Rizzi, Laia Querol Cano, Annemiek B. van Spriel

**Affiliations:** ^a^Department of Medical BioSciences, Radboud Institute for Medical Innovation, Radboud University Medical Center, Nijmegen 6525 GA, The Netherlands; ^b^Department of Chemistry, Structural Biochemistry, Bijvoet Centre for Biomolecular Research, Faculty of Science, Utrecht University Utrecht 3584 CH, The Netherlands; ^c^Radboud Institute for Medical Innovation, Radboud University Medical Center, Nijmegen 6525 GA, The Netherlands; ^d^Department of Molecular Immunology, Centre for Biological Signalling Studies and Centre for Integrative Biological Signalling Studies, Centre for Biological Signalling Studies, Biology III, Faculty of Biology, University of Freiburg, Freiburg 79104, Germany; ^e^Department of Rheumatology and Clinical Immunology, University Medical Center Freiburg, Faculty of Medicine, University of Freiburg, Freiburg 79106, Germany; ^f^Center for Chronic Immunodeficiency, University Medical Center Freiburg, Faculty of Medicine, University of Freiburg, Freiburg 79106, Germany; ^g^Department of Immunology, Leiden University Medical Center, Leiden 2333 ZG, The Netherlands; ^h^Oncode Institute, Leiden University Medical Center, Leiden 2333 ZG, The Netherlands; ^i^Department of Pediatric Immunology, Rheumatology and Infectious Diseases, Emma Children’s Hospital, Amsterdam University Medical Center, Amsterdam 1105 AZ, The Netherlands; ^j^Department of Hematology, Leiden University Medical Center, Leiden 2333 ZG, The Netherlands; ^k^Division of Clinical and Experimental Immunology, Institute of Immunology, Center for Pathophysiology, Infectiology and Immunology, Medical University of Vienna, Vienna 1090, Austria; ^l^Centre for Integrative Biological Signalling Studies, University of Freiburg, Freiburg 79104, Germany

**Keywords:** CD20, B lymphocytes, immune synapse, CD70

## Abstract

CD20 is a four-transmembrane protein expressed at the surface of B cells from late pro-B cells to memory B cells, with the exception of plasma cells. Its expression pattern makes it an attractive therapeutic target for different B cell malignancies and autoimmune diseases. Despite the clinical success of CD20-targeting antibodies, the biology of the CD20 protein is still not well understood. We investigated CD20 binding partners in the membrane of human B cells using immunoprecipitation followed by mass spectrometry analysis. We identified a molecular interaction between CD70 and CD20, and confirmed this using proximity ligation assays. CD20–CD70 spatiotemporal colocalization was validated at the plasma membrane of B cells using high-resolution microscopy. Cell surface expression of CD70 was found to be enhanced upon CD20 overexpression, suggesting a role for CD20 in stabilizing CD70 at the B cell membrane. Moreover, we observed impaired B-T cell synapse formation and defective recruitment of CD70 to the immunological synapse in the absence of CD20. Impaired synapse formation was confirmed by deleting CD20 in primary B cells, and analysis of B cells from a CD20-deficient patient. Finally, CD20-deletion resulted in diminished T cell activation and cytokine secretion. Together, this study demonstrates that CD20 interacts with CD70 at the B cell membrane, and that CD20 is required for immune synapse formation between B and T cells and consequent T cell activation.

## Significance

CD20, a membrane protein expressed on B cells, is a major therapeutic target in B cell malignancies and autoimmune diseases. Despite the clinical success of CD20-based immunotherapies, the function of CD20 is understudied. This study characterizes CD20 membrane organization and identifies the costimulatory molecule CD70 as a binding partner of CD20. We report a key role for CD20 in immune synapse formation between B and T cells, CD70 recruitment to the synapse and T cell activation. Thus, the clinical target CD20 is required for the interaction between B and T lymphocytes.

Antigen-presenting cells, including dendritic cells (DCs), B cells, and macrophages, scavenge for pathogens or aberrant cells and subsequently activate T cells to initiate adaptive immune responses (1). B cells can take up antigens through B cell antigen receptor (BCR) dependent or independent mechanisms. B cells employ the presentation of antigen-derived peptides by class II major histocompatibility complex (MHC) molecules to ensure high-affinity antibody production and antigen-specific T cell activation to generate cell-mediated immunity (2–4).

Antigen binding to the BCR and subsequent B cell activation results in antigen internalization, processing, and presentation on MHC II molecules, which is recognized by T cells through their T cell antigen receptor (TCR). Plasma membrane topography governs the three dimensional (3D) localization of Immunoglobulin M (IgM)-BCR clusters (5). Efficient B-T cell interactions require the formation of an immunological synapse (IS) or supramolecular activation cluster. The IS is a densely packed, highly dynamic, membrane structure that forms at the interface between B and T cells, and constitutes a signaling platform for T cell activation and proliferation (6–8). The IS ensures the timely localization of various membrane proteins and signaling molecules required for T cell activation in specialized membrane domains (8, 9), a process orchestrated by membrane organizers such as the actin cytoskeleton, lipid rafts, galectins, and tetraspanins (10–16). B cells recruit MHC and costimulatory molecules (such as CD80/86 and CD70) toward the IS. At the same time, T cells recruit the TCR and costimulatory molecules, most commonly members from the immunoglobin (Ig) superfamily (CD28), or from the tumor necrosis factor family (CD27) (17). CD27 is recruited to the T cell side of the IS where it interacts with its ligand CD70 on the B cell side. CD70, a homotrimeric type II transmembrane protein (18–21), is expressed on macrophages, mature DCs, natural killer (NK) cells as well as on activated B cells and T cells (22, 23). CD27 and CD28 have nonredundant costimulatory functions in T cells responsible for activating distinct signaling pathways. CD28 activates PI3K to regulate T cell costimulation, whereas CD27 induces TRAF activation thereby promoting T cell activation, IFN-γ secretion, and antitumor responses, indicating distinct roles in T cell immunity (22, 24). Upon DC maturation, CD70 traffics toward the cell surface via the MHC II compartment (MIIC), together with antigen-loaded MHC II (25). Patients with CD70 deficiency present a primary immunodeficiency associated with impaired terminal B cell development (26). However, little is known about the function and localization of CD70 upon antigen presentation by B cells.

CD20 (*MS4A1*), a member of the membrane-spanning 4A family, is expressed on the surface of most B cells, from pre-B cells to memory B cells, but is absent on plasma cells (27). Although CD20 shares some structural features with the superfamily of tetraspanin proteins (28), it lacks the tetraspanin-specific CCG motif in its large extracellular loop (29, 30). Interestingly, CD20 forms homotetramers at the plasma membrane, and has been found in complex with other membrane proteins including the BCR, MHC I, MHC II, and several tetraspanins (CD81, CD82, CD53, and CD37) (31–36). Functionally, CD20 has been shown to restrict the interaction of CD19 with the IgM-class BCR and thereby to block the transition from B cells to plasma cells (37). Importantly, CD20 is a major therapeutic target for mature B cell lymphomas (38), rheumatoid arthritis (39, 40), systemic lupus (41), and multiple sclerosis (42). However, lack of fundamental knowledge regarding the biological function of CD20 hampers the improvement of current CD20-targeting strategies in the clinic (36). The few reported interactions between CD20 and the BCR as well as MHC molecules, suggest a putative role for CD20 in B cell membrane organization and/or the antigen presentation machinery of B cells.

Here, we characterized the basal biophysical properties of CD20 at the B cell surface, revealing its clustered organization and lateral mobility. Through coimmunoprecipitation coupled with mass spectrometry (MS) analysis, we identified CD70 as a CD20 binding partner in B cells. We observed impaired synapse formation and CD70 recruitment toward the IS in B cells lacking CD20, which also diminished subsequent T cell activation and cytokine secretion.

## Results

### CD20 Is Highly Clustered at the Cell Membrane.

**Fig. 1.**
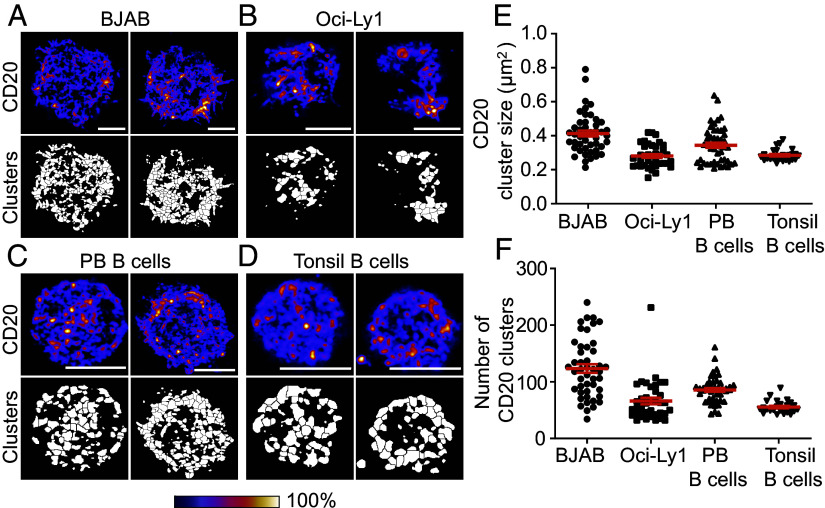
CD20 is highly clustered at the cell surface of B cells. (*A*–*D*) Representative Airyscan confocal images of CD20 staining (*Top*), expression intensity shown by color gradient (bottom bar), and defined CD20 clusters (*Bottom*), for BJAB (*A*), Oci-Ly1 (*B*), PB B cells (*C*) and tonsil B cells (*D*). (Scale bar: 5 μm.) (*E* and *F*) Quantification of CD20 cluster size in μm^2^ (*E*) and the number of CD20 clusters (*F*) defined on the bottom of the plasma membrane for BJAB, Oci-Ly1, PB, and tonsil B cells. Datapoints are 46 (BJAB), 38 (Oci-Ly1), 47 (PB B cell), and 25 (Tonsillar B cells) cells derived from 4 (BJAB), 2 (Oci-Ly1), 3 (PB), and 1 (tonsil) independent experiments or donors. Data represent mean ± SEM.

To gain insights into the biological function of CD20, we first investigated the spatial distribution of CD20 on lymphoma B cell lines and primary B cells using high-resolution microscopy. We detected that CD20 is clustered on the cell surface of BJAB and Oci-Ly1 cell lines (Fig. 1 *A* and *B*) and confirmed these findings on primary B cells isolated from peripheral blood (PB) and tonsils (Fig. 1 *C* and *D*). Next, the number and size of CD20 clusters at the membrane of B cell lines and primary B cells were quantified (Fig. 1 *E* and *F* and *SI Appendix*, Fig. S1 *A* and *B*). The average number of CD20 molecules per cell and per cluster was similar between the cell lines and primary B cells (*SI Appendix*, Fig. S1*C*). Overall, these data demonstrate the clustered nature of CD20 at the B cell surface.

**Fig. 2.**
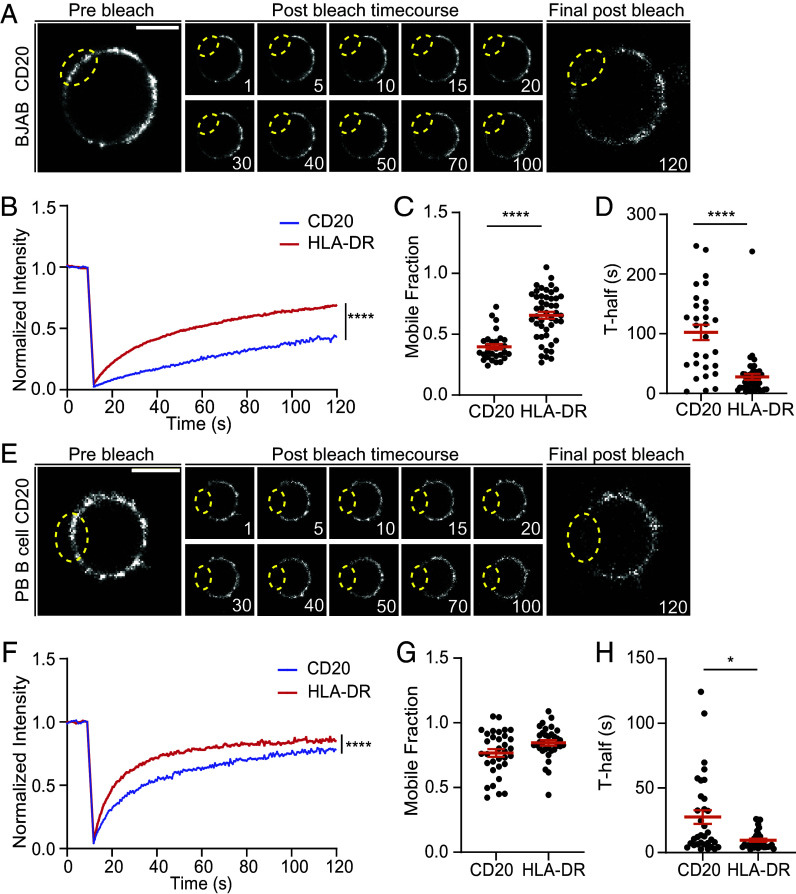
CD20 is mobile at the B cell surface. (*A*) Representative confocal images of CD20 FRAP time course in BJAB cells. Images show CD20 signal and distribution prebleach (*Left*), and postbleach imaging (*Middle*) and final post bleach image (*Right*). The yellow circle indicates the bleached area and timing (seconds) as indicated in figure. (Scale bar: 5 μm.) (*B*) Mean recovery curves of CD20 and HLA-DR (positive control) signal normalized to prebleach intensity in BJAB cells. (*C*) Quantification of CD20 and HLA-DR mobile fractions in BJAB cells. (*D*) CD20 recovery speed indicated by T-half in seconds in BJAB cells. (*E*) PB B cells were analyzed as described in A. (Scale bar: 5 μm.) (*F*) Mean recovery curves of CD20 and HLA-DR (positive control) signal normalized to prebleach intensity in PB B cells. (*G*) Quantification of CD20 and HLA-DR mobile fractions in PB B cells. (*H*) CD20 recovery speed indicated by T-half in seconds in PB B cells. Datapoints are 30 (CD20, BJAB), 47 (HLA-DR, BJAB), or 33 (PB B cells) cells derived from three (CD20, BJAB), four (HLA-DR, BJAB), or three (PB B cells) independent experiments or donors. Statistical significance was assessed by the Mann–Whitney *U* test, mean ± SEM is shown (*P < 0.05, ****P < 0.0001).

Next, we investigated the lateral mobility of CD20 at the plasma membrane of BJAB cells and primary B cells using fluorescence recovery after photobleaching (FRAP). Time-lapse imaging showed CD20 recruitment into the bleached area (Fig. 2 *A* and *E*) albeit less than that observed for HLA-DR (*SI Appendix*, Fig. S2 *A* and *B*), used as a positive control (43), as shown in the recovery curves (Fig. 2 *B* and *F*). This was confirmed by quantifying the CD20 mobile fraction and its half-time of recovery (T-half) in BJAB and PB B cells (Fig. 2 *C*, *D*, *G*, and *H*). To further characterize CD20 membrane dynamics, we performed single-particle tracking using total internal reflection fluorescence (TIRF) microscopy in BJAB and PB B cells (*SI Appendix*, Fig. S2 *C*–*I*). CD20 molecules switched between active and confined movement behavior, and displayed an average speed of ~0.15 µm/s, demonstrating CD20 mobility at the cell surface of B cells.

### CD70 Is a Binding Partner of CD20.

**Fig. 3.**
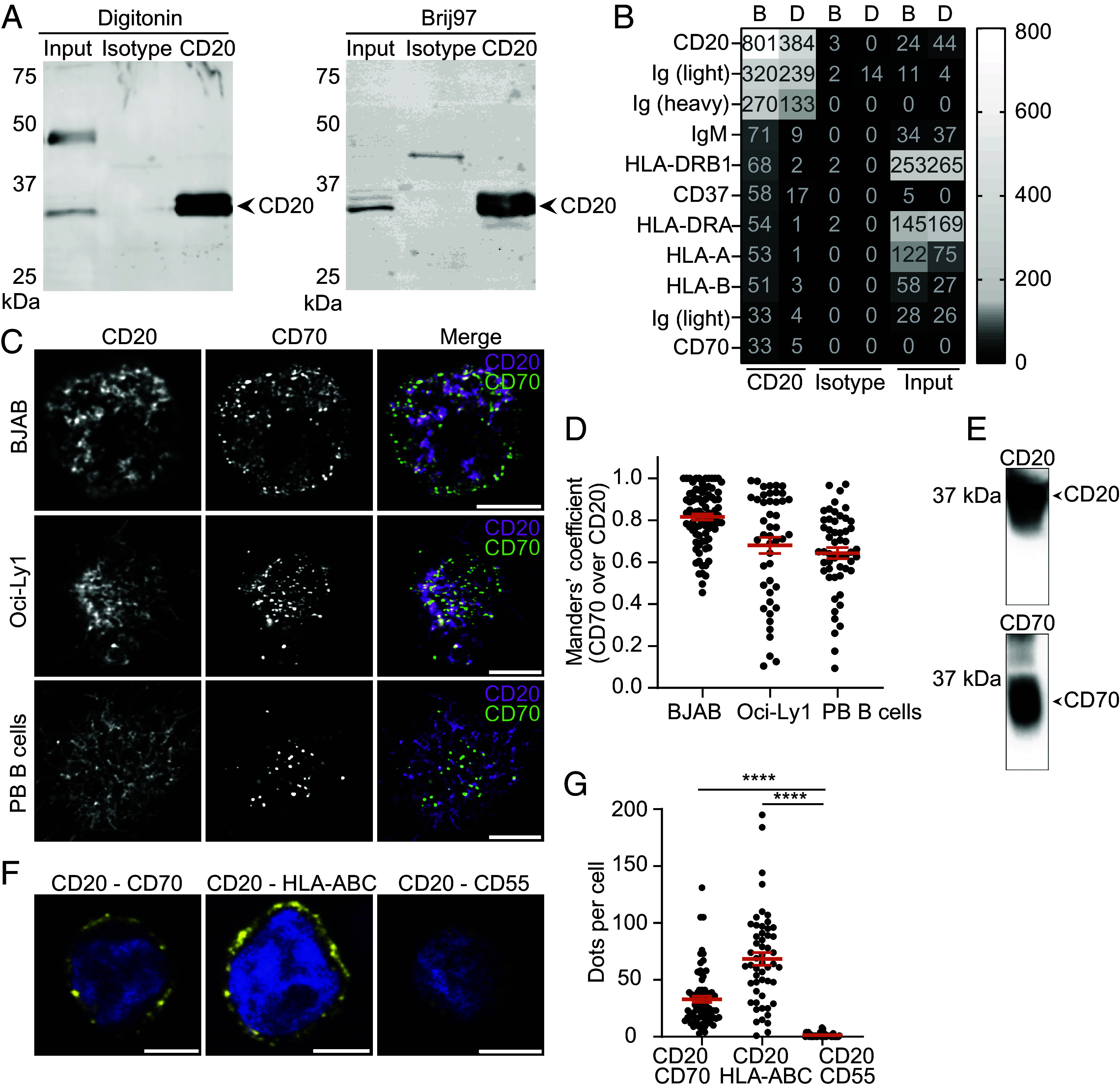
CD20 interacts with CD70 at the cell surface of B cell lines. (*A*) IP of CD20 using detergents Digitonin (*Left*) and Brij97 (*Right*). (*B*) Heatmap of iBAQ values showing the highest enriched proteins hits detected by MS upon CD20 Co-IP using Brij97 (B) or Digitonin (D). Enrichment intensity shown by gradient scale (right bar). (*C*) Representative confocal microscopy images of CD20 (*Left* column, magenta) and CD70 (middle column, green) and the merged image (*Right* column) in BJAB (*Top* row), Oci-Ly1 (*Middle* row), and PB B cells (*Bottom* row). (Scale bar: 5 μm.) (*D*) Manders’ overlap coefficient (M1) of CD70 overlap over CD20 for BJAB, Oci-Ly1, and PB B cells. Datapoints derived from four (BJAB), two (Oci-Ly1), and three (PB B cells) independent experiments or donors. Mean ± SEM shown. (*E*) Copurification of CD20 (*Left*) and CD70 (*Right*) in HEK293 cells. (*F*) Representative confocal microscopy images of in situ PLA on BJAB cells stained for CD20 and CD70 (*Left*), CD20 and HLA-ABC (*Middle* panel, positive control), and CD20 and CD55 (*Right* panel, negative control). (Scale bar: 5 μm.) (*G*) Dots per cell were quantified from three independent experiments (CD70 n = 74, HLA-ABC n = 53, and CD55 n = 56 cells). Data represent mean ± SEM. Statistical significance was assessed by one-way ANOVA with Tukey’s post hoc test. (****P < 0.0001).

To gain mechanistic insights into the spatiotemporal localization of CD20, we investigated CD20 interacting proteins at the plasma membrane of B cells by immunoprecipitation (IP) of endogenous CD20 protein followed by MS (Fig. 3 *A* and *B*, *SI Appendix*, Fig. S3*A*, and Dataset S1). CD20 was enriched in the CD20 IP compared to input and isotype control, both in the western blot (Fig. 3*A* and *SI Appendix*, Fig. S3*A*) and MS results (Fig. 3*B*), confirming the quality of the pulldown experiment. Known CD20 binding partners were identified by MS, including the IgM-class BCR (31), MHC I (HLA-A and -B) (32), MHC II (HLA-DRB1 and -DRA) (32, 33), and tetraspanin CD37 (34) validating the co-IP/MS strategy (Fig. 3*B*). Interestingly, costimulatory molecule CD70 was identified among the highest enriched proteins in the IPs under both mild (Brij97) and more stringent (digitonin) conditions (Fig. 3*B*). Next, colocalization studies were performed to assess the spatiotemporal distribution of both CD20 and CD70 at the B cell membrane. We identified a clear overlap between CD20 and CD70 at the cell surface of both BJAB, Oci-Ly1, and PB B cells (Fig. 3*C*) which was quantified using Manders’ overlap coefficient (Fig. 3*D*) and Pearson’s correlation coefficient (*SI Appendix*, Fig. S4 *A*–*C*). CD20 colocalization with HLA-DR (32, 33) or with the GPI-linked protein CD55 (44) was determined as positive and negative control in these experiments. CD70 colocalized with CD20 significantly higher than with CD55 using both Pearson’s and Manders’ coefficient analysis, confirming the CD20–CD70 association (*SI Appendix*, Fig. S4 *D*–*F*). CD20–CD70 interaction was confirmed by copurification of CD20 and CD70 after transient transfection in HEK293 cells (that do not contain endogenous CD20 and CD70) followed by purifying CD70 using a Strep II tag via a Streptactin resin. Successful copurification of CD20-His was confirmed via immunological detection of the His-tag in the CD70 elution fractions (Fig. 3*E* and *SI Appendix*, Fig. S3*B*). Next, we also validated CD20–CD70 interaction in intact membranes using proximity ligation assays (PLA), that allows for in situ detection of endogenous protein interactions within nanometer distance (45). A specific signal indicative of interaction between CD20 and CD70 was observed (Fig. 3 *F* and *G*). HLA-ABC (MHC I), a known binding partner of CD20 (32), was used as positive control, whereas CD55 (44) served as negative control (Fig. 3 *F* and *G*). In summary, these data identify CD70 as binding partner for CD20 on B cells and confirm their spatial colocalization at the B cell surface.

### CD20 Stabilizes CD70 at the B Cell Surface.

**Fig. 4.**
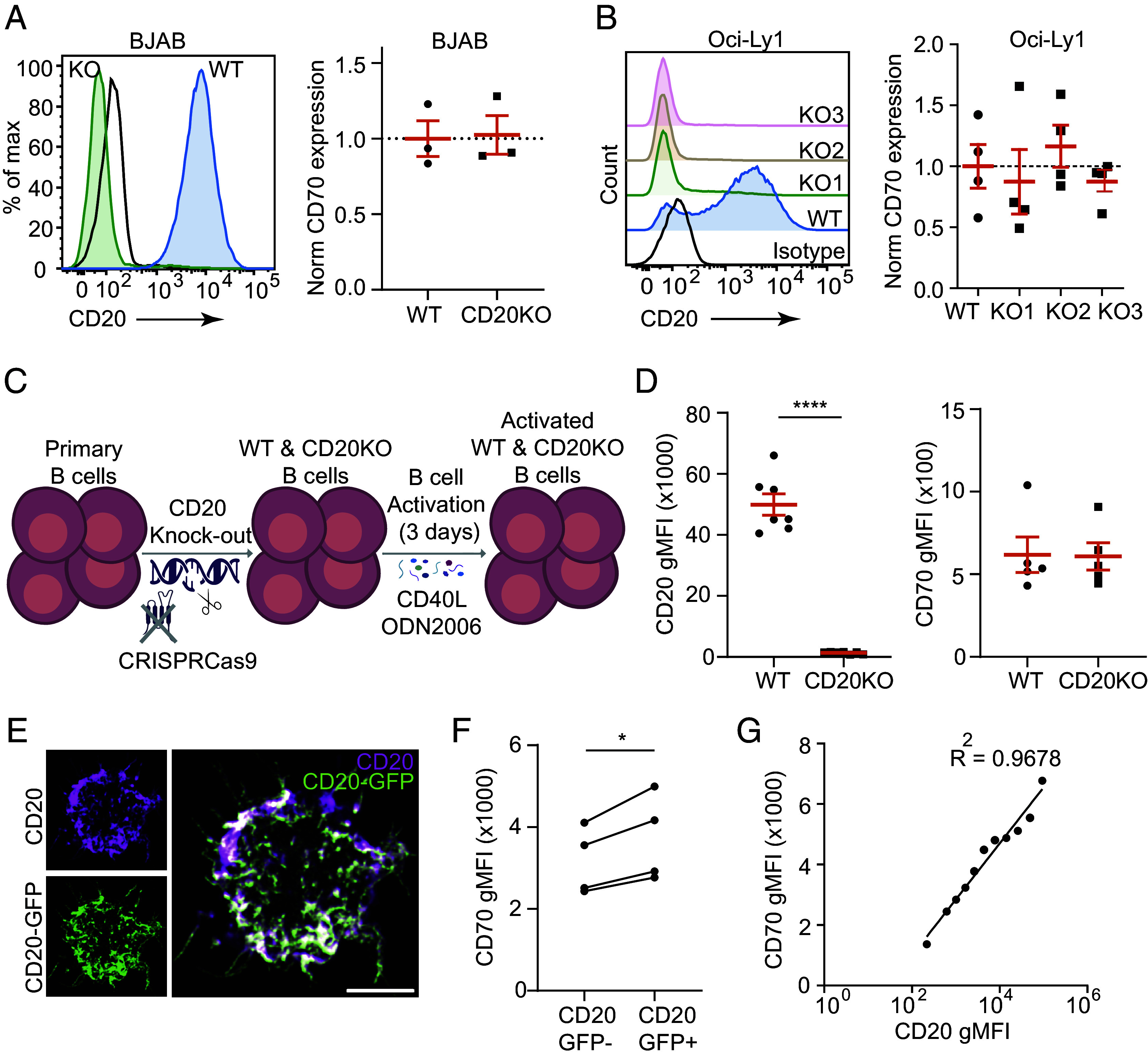
Effect of CD20 on CD70 cell surface expression in B cell lines and primary B cells. (*A*) CD20 and CD70 surface expression in WT and CD20KO BJAB cells. Representative flow cytometry histogram showing CD20 expression in WT (blue) and CD20KO (green) BJAB cells (*Left*). Isotype control is shown in black. Quantification of normalized CD70 MFI values in WT and CD20KO BJAB cells (*Right*). Datapoints derived from three independent experiments. (*B*) CD20 and CD70 surface expression in WT and CD20KO Oci-Ly1 cells. Representative flow cytometry histogram showing CD20 expression in WT (blue) and CD20KO1 (green), CD20KO2 (orange), and CD20KO3 (pink) Oci-Ly1 cells (*Left*). Isotype control is shown in black. Quantification of normalized CD70 MFI values in WT and CD20KO Oci-Ly1 cells (*Right*). Datapoints derived from four independent experiments. (*C*) Schematic representation of the generation of CD20KO primary B cells. (*D*) CD20 (*Left*) and CD70 (*Right*) surface expression in WT and CD20KO activated primary B cells by flow cytometry (seven donors). (*E*) Representative confocal image of BJAB WT cells expressing CD20-GFP (green, *Bottom Left*) and stained for CD20 (endogenous and exogenous) (magenta, *Top Left*). Merged image (*Right*) shows overlap. (Scale bar: 5 μm.) (*F*) Quantification of normalized CD70 MFI values in BJAB cells transfected with CD20-GFP, gated for GFP^+^ or GFP^−^. Datapoints derived from four independent experiments. (*G*) Correlation between CD20 and CD70 surface expression in CD20-GFP-positive BJAB cells. Statistical significance was assessed by the Mann–Whitney *U* test (*A*), Friedmann test followed by Dunn’s multiple comparison test (*B*), or paired *t* test (*D* and *F*). (*P < 0.05, ****P < 0.0001). All graphs depict mean values ± SEM.

Next, we examined the molecular and functional relation between CD20 and CD70 by investigating CD20 knockout (CD20KO) BJAB (Fig. 4*A*) and three independent CD20KO Oci-Ly1 (Fig. 4*B*) cell lines generated by CRISPR/Cas9 technology. No significant differences in endogenous CD70 surface expression levels were observed between the CD20KO BJAB (Fig. 4*A*), CD20KO Oci-Ly1 (Fig. 4*B*), and CD20-expressing wild type (WT) counterparts. Next, we deleted CD20 in primary B cells using CRISPR/Cas9 followed by CD40L and ODN2006 stimulation to promote B cell survival and upregulate CD70 expression. This resulted in very efficient knockout of CD20 (Fig. 4 *C* and *D*: seven donors), which did not affect expression of CD70 (Fig. 4*D* and *SI Appendix*, Fig. S5). Similarly, CD20 loss did not affect CD70 membrane organization as no differences in the number, size, or intensity of CD70 surface clusters were observed between CD20KO BJAB cells and their WT counterparts (*SI Appendix*, Fig. S6). In addition, the effect of CD20 overexpression on CD70 protein levels was analyzed in BJAB WT cells expressing exogenous CD20-GFP or GFP as control. We verified that CD20-GFP showed comparable cell surface localization and expression to endogenous CD20, as indicated by the clear overlap of CD20-GFP and CD20 antibody signal (Fig. 4*E* and *SI Appendix*, Fig. S7*A*). CD20 expression levels in BJAB cells were comparable to those in primary B cells, whereas CD20 overexpression (CD20-GFP) led to slightly higher CD20 surface expression (*SI Appendix*, Fig. S7*B*). Interestingly, we detected a significant increase in CD70 membrane expression in CD20-GFP-positive cells compared to CD20-GFP negative cells (Fig. 4*F*). Moreover, CD20 and CD70 surface levels positively correlated in CD20-GFP transfected cells (Fig. 4*G*). These findings support that while CD20 is not essential for CD70 expression on the membrane of B cells, it may stabilize CD70 at the B cell surface. To study whether CD70 was important for the expression of CD20, CD70KO BJAB cells were generated (*SI Appendix*, Fig. S8*A*). No significant differences in endogenous CD20 surface expression were observed between CD70KO BJAB cells and the CD70-positive parental cells (*SI Appendix*, Fig. S8*B*), and CD70 overexpression did not affect endogenous CD20 protein levels in BJAB cells (*SI Appendix*, Fig. S8*C*). These findings indicate that CD70 is not required for the expression of CD20 at the B cell surface. Moreover, CD20 mobility did not depend on CD70, as both the mobile fraction and recovery curves of CD20 were comparable between WT and CD70KO BJAB cells (*SI Appendix*, Fig. S8 *D*–*G*).

### CD20 Does Not Influence CD70 Mobility or Recruitment to Ligand CD27 in Steady State.

Next, we investigated whether CD20 loss affected CD70 mobility and recruitment toward its natural ligand CD27. FRAP studies on WT and CD20KO BJAB cells expressing CD70-GFP showed clear lateral movement of CD70 in both WT and CD20KO B cells (*SI Appendix*, Fig. S9 *A* and *B*). However, CD20 loss did not result in altered CD70 mobility, as both the mobile fraction and recovery curves of CD70-GFP were comparable between WT and CD20KO B cells (*SI Appendix*, Fig. S9 *C* and *D*). In addition, we employed microcontact printing (46, 47) to study CD70 mobility upon CD27 ligand binding in B cells lacking CD20. This live cell imaging technique allows for analysis of directed CD70 recruitment toward specific areas enriched for CD27 by means of microcontact printing of recombinant CD27, creating localized ligand-enriched regions for receptor interaction studies (46, 47). WT and CD20KO B cells were transfected with CD70-GFP and seeded on surfaces containing localized CD27 stamps, after which localization of CD70 was assessed (*SI Appendix*, Fig. S9 *E* and *F*). We observed specific CD70-GFP recruitment on CD27^+^ stamps, which was similar between WT and CD20KO B cells (*SI Appendix*, Fig. S9 *G* and *H*). Combined, these data indicate that CD20 does not affect CD70 mobility in steady state.

### Synapse Formation and CD70 Recruitment Are Abrogated by CD20 Loss.

**Fig. 5.**
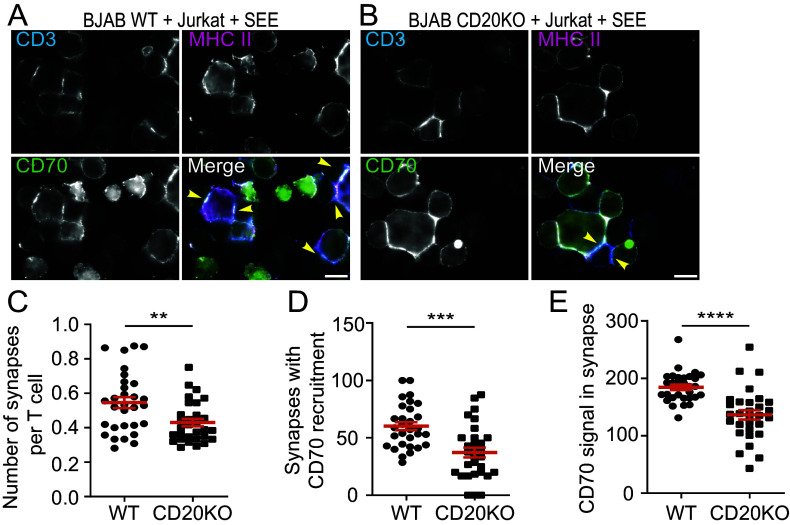
CD20 deletion in BJAB cells results in defective IS formation and less CD70 recruitment toward the synapse. (*A* and *B*) Representative epifluorescence images of cocultures with Jurkat and WT (*A*) or CD20KO (*B*) BJAB cells pulsed with SEE. Cells were stained against CD3 (blue, *Top Left*), MHC II (magenta, *Top Right*), and CD70 (green, *Bottom Left*). *Bottom Right* shows merged panel and yellow arrows indicate immune synapses. (Scale bar: 10 μm.) (*C*) Quantification of mean number of synapses per Jurkat T cell in cocultures with WT and CD20KO BJAB cells. (*D*) Quantification of synapses with 1.5 times enriched CD70 intensity in CD3-enriched areas as percentage of total synapses in cocultures between Jurkat T cells and WT or CD20KO BJAB cells. (*E*) Quantification of CD70 intensity in CD3-enriched areas as a percentage of mean CD70 intensity in cocultures of Jurkat T cells and WT or CD20KO BJAB cells. Graphs show mean ± SEM values from three independent experiments. 30 images per condition (WT or CD20KO) were analyzed, ranging between 300 to 400 synapses analyzed per condition. Statistical significance was assessed by the Mann–Whitney *U* test (*C* and *D*) or unpaired *t* test (*E*), (**P < 0.01, ***P < 0.001, ****P < 0.0001).

**Fig. 6.**
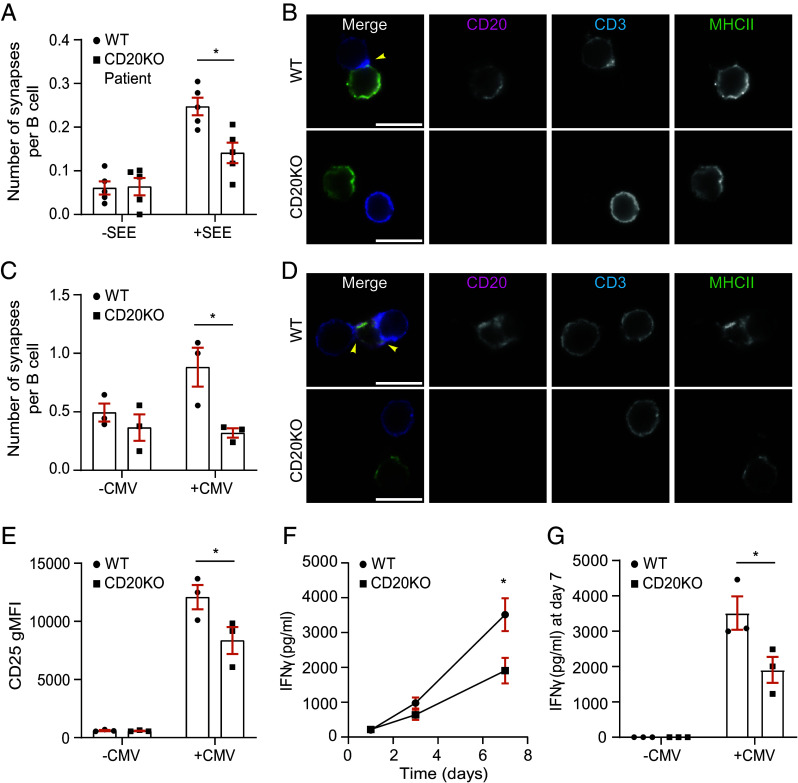
CD20 deficiency in primary B cells impairs synapse formation, affecting T cell activation and cytokine secretion. (*A*) Quantification of number of synapses per B cell in cocultures of primary T cells with activated primary B cells from healthy donors (WT) or a CD20-deficient patient (CD20KO), with/without SEE. Datapoints derived from five donors and two independent experiments. 85 (-SEE, WT), 90 (-SEE, CD20KO), 93 (+SEE, WT), and 90 (+SEE, CD20KO) images were analyzed. (*B*) Representative epifluorescence zoomed images of these cocultures stimulated with SEE. Shown are merged images (*Left*), CD20 (magenta, *Middle Left*), CD3 (blue, *Middle Right*), and HLA-DR (green, *Right*). Yellow arrows indicate immune synapses. (Scale bar: 10 μm.) (*C*) Quantification of number of synapses per B cell in cocultures of CMV-specific CD4+ T cells with WT or CD20KO activated primary B cells with/without CMV peptide. Datapoints derived from three donors. 67 (-CMV) and 72 (+CMV) images were analyzed. (*D*) Representative epifluorescence zoomed images of these cocultures stimulated with CMV. Shown are merged images (*Left*), CD20 (magenta, *Middle Left*), CD3 (blue, *Middle Right*), and HLA-DR (green, *Right*). Yellow arrows indicate immune synapses. (Scale bar: 10 μm.) (*E*) Quantification of CD25 expression in CMV-specific CD4+ T cells after 24 h of coculture with WT or CD20KO primary B cells, with/without CMV. (*F* and *G*) Quantification of IFNγ secretion by these CMV-specific CD4+ T cells in time (*F*) and quantification at day 7 (*G*). Datapoints derived from three donors. Statistical significance was assessed by two-way ANOVA with Sidak’s multiple comparisons test (*A*, *C*, *F*, and *G*), or one-tailed unpaired t test with Welch’s correlation (*E*). (*P < 0.05). Mean ± SEM is shown.

In DCs CD70 localizes toward the IS in an MHC II-dependent manner (25), promoting T cell activation and survival upon CD27 ligation (22, 24, 48, 49). We determined the effect of CD20 loss in a B-T cell synapse model using WT or CD20KO BJAB cells that were cultured with Jurkat T cells and superantigen staphylococcal enterotoxin E (SEE). CD3 enrichment at the contact point in Jurkat cells was observed when incubated with WT or CD20KO BJAB cells (Fig. 5 *A* and *B* and *SI Appendix*, Fig. S10 *A* and *B*: yellow arrows), but not in the absence of SEE (*SI Appendix*, Fig. S10 *C* and *D*). Strikingly, we detected a significant decrease in synapse formation when CD20 was absent in B cells (Fig. 5 *A*–*C* and *SI Appendix*, Fig. S10 *A* and *B*: yellow arrows). In addition, CD70 recruitment to the IS was impaired in CD20KO B cells compared to WT cells, demonstrated by both the number of synapses showing CD70 recruitment, and by the overall CD70 signal within the synapse (Fig. 5 *D* and *E*). No differences in MHC II recruitment to the synapse and surface expression (*SI Appendix*, Fig. S10 *E* and *F*) were observed between CD20KO and WT BJAB cells, indicative of a direct effect of CD20 on CD70 recruitment to the IS. We validated these results in experiments with purified primary B cells from a CD20-deficient patient (CD20KO, *SI Appendix*, Fig. S11*A*) (50). Absence of CD20 did not alter CD70 or HLA-DR surface expression (*SI Appendix*, Fig. S11 *B* and *C*), in line with CRISPR CD20KO in primary cells (Fig. 4*D*). However, B cells from a CD20-deficient patient showed defective synapse formation compared to B cells isolated from healthy donors (WT) (Fig. 6 *A* and *B*). We further validated the importance of CD20 in synapse formation using a mixed lymphocyte reaction model, in which we cocultured activated WT or CD20KO primary B cells with allogeneic primary T cells (*SI Appendix*, Fig. S12). Next, we established an antigen-specific synapse model using human primary B and T cells. Activated WT or CD20KO primary B cells were cocultured with primary CMV-pp65-specific CD4^+^ T cells (51, 52) and synapse formation was analyzed by epifluorescence microscopy. WT B cells pulsed with CMV peptide showed clear synapse formation with CMV-specific T cells, which was abrogated in the absence of CD20 (Fig. 6 *C* and *D*). Next, we analyzed T cell activation and cytokine secretion in this CMV-antigen model. Importantly, T cells cocultured with CD20KO B cells displayed lower CD25 expression, a marker of T cell activation (Fig. 6*E*), and reduced IFN-γ (Fig. 6 *F* and *G*) and IL-2 (*SI Appendix*, Fig. S11*D*) secretion. Collectively, these data demonstrate that CD20 is required for proper synapse formation by B cells, and directly contributes to subsequent T cell activation and cytokine secretion.

## Discussion

CD20, a membrane protein expressed on the surface of most B cells (27), represents a key therapeutic target for B cell lymphomas (38). CD20 has been shown to interact with other membrane proteins including the BCR, MHC I and II, and tetraspanins (31–35) and to restrict CD19 interaction with the BCR, impeding B cell differentiation into plasma cells (37). Nonetheless, the biological functions of CD20 are still poorly described and its biophysical characteristics at the B cell membrane have not been resolved. The organization of membrane proteins is crucial for various cellular processes, with many being organized into specialized domains that form signaling platforms, such as the IS between lymphocytes (8). In this study, we show that CD20 is highly clustered at the B cell membrane, exhibiting both mobile and immobile populations. CD20 mobility may allow for binding different interaction partners (BCR, MHC molecules, tetraspanins) (31–35) depending on the activation state of the B cell.

We identified the costimulatory molecule CD70 as a binding partner of CD20. At the IS, CD70 expressed by B cells binds CD27 at the T cell membrane, in turn triggering c-Jun pathway signaling via TRAF2/5 leading to T cell activation (22, 24, 48, 49). CD70 exists as a trimer at the cell surface in steady state, and oligomerization of CD70 trimers is necessary to fully access signaling through the CD27/CD70 axis (20, 53, 54). We observed CD20 to be dispensable for clustering of CD70 at the resolution limit of the Airyscan microscope (150 nm), although effects on smaller scale cannot be excluded. CD70 ligation in B cells induces B cell expansion through the activation of the PI3K/Akt and MEK signaling pathways, a process referred to as reverse signaling (22, 55). In addition, CD70 has been postulated to either induce (22, 55, 56) or inhibit (57, 58) B cell differentiation through this reverse signaling. In hematological malignancies, signaling through the CD27/CD70 axis stimulates cell survival, proliferation, and the acquisition of stemness properties in malignant cells, thereby contributing to disease progression (55). We demonstrate that CD20 is required for proper IS formation between B and T cells and CD20 loss resulted in defective CD70 recruitment to the IS. Consequently, CD20 loss negatively impacted T cell activation, illustrated by lower surface expression of the activation marker CD25 on T cells and reduced cytokine production. We confirmed these findings in primary cells, involving a peptide MHC-dependent IS between B cells and antigen-specific T cells. The humoral immunodeficiency observed in the CD20-deficient patient (50) may be linked to impaired synapse formation in vivo. Our finding that CD20 overexpression led to enhanced CD70 surface levels indicates that CD20 stabilizes CD70 in the plasma membrane. The CD20–CD70 interaction was also preserved in cells that do not endogenously express these proteins (HEK293), indicating a strong molecular interaction that does not depend on other CD20-partners within B cells (BCR, MHC molecules, tetraspanins) (31–35). The PLA combined with strong co-IP data support a direct molecular interaction and future studies are needed to resolve the 3D structure of this complex.

In DCs, CD70 localization toward the IS depends on MHC II recruitment (25). Antigen-loaded MHC II normally traffics toward the cell surface via the MIIC (59), and CD70 is reported to traffic together with tetraspanin CD63 as part of the MIIC toward the membrane upon DC maturation (25, 60). However, we observed that in B cells the impairment of CD70 recruitment toward the IS upon CD20 loss was independent of MHC II, as MHC II recruitment toward the synapse was normal in CD20KO B cells. In addition, the effect of CD20 on CD70 recruitment toward CD27 did not occur at a steady state, but rather upon IS formation with T cells. Therefore, we hypothesize that once CD70 has been translocated to the B cell membrane with the MIIC complex, the multiple spatial rearrangements that take place during IS formation may induce additional signals, leading CD20 to act as an anchor for CD70, and thereby ensure its presence at the synapse. Together, our findings indicate a role for CD20 on the CD27/CD70 signaling axis during antigen presentation, and thus, on B and T cell activation and differentiation.

Differential CD70 recruitment to the IS might determine the antigen presentation capabilities of B cells and DCs. Notably, B cells and DCs differ in their interaction dynamics with T cells, with B cells displaying more rapid, transient interactions (4). Interestingly, costimulation through CD70 has been described to be involved in antiviral and antitumor immunity, both requiring cytotoxic T cell (CTL) activation (25, 48, 49, 61, 62) and murine T helper CD4^+^ cells activate cytotoxic CD8^+^ CTLs via the CD27/CD70 axis (56, 63–65). Existing literature has predominantly focused on CTL activation by DCs whereas the role of B cells in this process has been largely overlooked (66). Further research could explore differences in T cell effector functions induced by DCs or B cells and how CD70-CD20 impacts these processes.

In conclusion, we report that CD20 is required for the establishment of the IS between B and T cells and subsequent T cell activation, and identified CD70 as a binding partner of CD20. Our findings highlight a function of CD20 as a membrane organizer during antigen presentation, thus aiding in further unraveling CD20 functions. Given that CD20 is a prominent therapeutic target in B cell lymphomas, CD20 membrane organization and mobility may not only influence its interactions with other membrane proteins, but also affect the accessibility of CD20-targeting antibody therapies. Interestingly, rituximab binding to CD20 has been shown to preserve CD20 oligomeric state (67). Gaining fundamental knowledge on CD20 biology is crucial for understanding and overcoming treatment resistance to CD20 immunotherapy.

## Materials and Methods

All antibody details are listed in *SI Appendix, Table*.

### Isolation of Primary B and T Cells from PB and tonsils.

Human PB lymphocytes were isolated from buffy coats obtained from healthy volunteers with approved study protocols of Sanquin blood bank, and in accordance with the recommendations of the institutional guidelines (Radboudumc, Nijmegen, The Netherlands). All subjects gave written informed consent in accordance with the Declaration of Helsinki. To isolate tonsillar B cells, tonsils were cut in half and lymphocytes were pushed out of the tissue using a 70 µm filter, while rinsing with Rosewell Park Memorial Institute (RPMI) medium 1640 (Gibco) with 10% fetal bovine serum (FBS; Hyclone) and 1% antibiotic-antimycotic (Gibco). Red blood cells were lysed using ammonium-chloride–potassium lysis buffer (made in house) for 2 min on ice. Primary B and T cells were isolated from PB and tonsil single-cell suspensions using Pan B or Pan T magnetic-activated cell sorting isolation kit (Miltenyi) according to the manufacturer’s protocol, and as previously described (68). When appropriate, primary B cells were activated with 2 µg/mL CD40L (Biolegend) and 1 µM of ODN2006 (Invivogen) in Iscove’s Modified Dulbecco’s Medium (IMDM; Gibco) with 1:100 Insulin-Transferrin-Selenium (100×), 1:100 non-essential amino acids (NEAA), and 10 µM of beta-mercaptoethanol (Thermo Fisher) with 10% FBS for 3 d.

### Culture of B Cell Lines.

Human lymphoma BJAB (Cat: ACC 757) and Oci-Ly1 (Cat: ACC 722) cells were maintained at 37 °C in 5% CO_2_. Cells were cultured in growth media RPMI 1640 with 10% (Oci-Ly1) or 20% (BJAB) FBS, 1% stable glutamine (Capricorn Scientific), and 1% antibiotic-antimycotic (Gibco). Cell lines were derived from Deutsche Sammlung von Mikroorganismen und Zellkulturen and authenticated using STR analysis.

### Generation of CD20 or CD70 Knock-Out (KO) Cell Lines or Primary B Cells by CRISPR/Cas9.

CD20KO BJAB cells were generated as previously reported (37). To generate CD20KO Oci-Ly1 cells, CRISPR/Cas9 deletion was carried out using guide RNA pairs targeting the third exon of the human CD20 gene (37). 2.5 × 10^6^ Oci-Ly1 cells were transfected with 4 µg of guide RNA (gRNA) plasmid in transfection media (Lonza) with a Nucleofector 4D system (Amaxa, Lonza) using program DN-100. After transfection, cells were cultured at 37 °C and 5% CO_2_. GFP-positive cells were flow sorted on a FACSAria instrument (BD Biosciences) 24 h posttransfection. GFP+ cells were sorted and plated as a single-cell suspension, to grow individual CD20KO clones. After expanding the Oci-Ly1 sorted culture for a week, cells were stained for CD20 with a Fluorescein Isothiocyanate labeled antibody, and CD20-negative cells were flow sorted.

KO of CD20 in primary B cells was obtained using CAS9NLS (Horizon) and a guideRNA (Horizon) for CD20 (Edit-R predesigned synthetic single guide RNA (sgRNA) MS4A1 target sequence GTCTTCTGATGATCCCAGCA). Prior to the nucleofection of the B cells, CAS9NLS was incubated with the sgRNA to form the complex for 20 min at room temperature (RT). The CAS9NLS/sgRNA complex was nucleofected with the Neon electroporation system (Thermo Fisher) (2,350 V, 20 ms, 1 pulse). For this 0.5*10^6^ cells were resuspended in 9 µL T-buffer and gently mixed with 8.5 µM CAS9NLS, 10 µM sgRNA complex, and 1.8 µM Electroporation Enhancer (IDT). After the nucleofection, cells were added to a round bottom 96-well plate in IMDM containing 1:100 Insulin-Transferrin-Selenium (100×), 1:100 NEAA, and 10 µM of β-mercaptoethanol (Thermo Fisher). After 2 h resting, IMDM containing 10% FBS and 10 ng/mL of BAFF (Miltenyi) was added. The next day, cells were activated with IMDM supplemented with 1:100 Insulin-Transferrin-Selenium (100×), 1:100 NEAA, 10 µM of beta-mercaptoethanol, 10% FBS, 2 µg/mL CD40L (Biolegend) and 1 µM ODN2006 (Invivogen). Cells were analyzed after 3 d of incubation.

To generate CD70KO BJAB cells, guide RNA pair targeting the first exon of the human CD70 gene were designed using CRISPOR.org (69). The guide RNAs were generated with the relevant oligos, ordered from Merk Sigma-Aldrich (oligo sense: CACC**GCTTTGGTCCCATTGGTCGC**, oligo antisense: AAAC**GCGACCAATGGGACCAAAG**C) and cloned into the PX458 Cas9 vector (Addgene). 5 × 10^6^ BJAB cells were transfected with 4.0 µg of gRNA plasmid in transfection media (Lonza) using a Nucleofector 4D system (Amaxa, Lonza), using program DS-104. One day after nucleofection, GFP-positive cells were flow sorted on a FACSAria instrument (BD Biosciences). After expanding the sorted culture for a week, cells were stained for CD70 with an AF647-labeled antibody, and CD70-negative cells were flow sorted.

### Flow Cytometry.

Cell suspensions containing 2 × 10^5^ cells were blocked with PBA [phosphate-buffered saline (PBS) + 1% bovine serum albumin (BSA) + 0.05% Sodium Azide] supplemented with 2% human serum and 1% goat serum (blocking buffer) for 30 min at 4 °C. Primary antibodies were incubated for 25 min at 4 °C, washed with blocking buffer and incubated with secondary antibodies for 25 min at 4 °C. Cells were washed in PBS and analyzed on a FACSVerse or FACSLyric flow cytometer (BD Biosciences), and data were analyzed using FlowJo X Software (FlowJo LLC).

### Immunofluorescence Microscopy.

Cell suspensions containing 2 × 10^5^ cells were blocked for 30 min at 4 °C with PBS containing 3% BSA, 2% human serum, and 1% goat serum. Primary antibodies were incubated for 25 min at 4 °C, washed with blocking buffer and incubated with respective secondary antibodies for 25 min at 4 °C when necessary. Next, cells were washed with PBS and adhered on Poly-L-lysine (PLL)-coated coverslips for 15 min at RT. Cells were then fixed in 4% paraformaldehyde (PFA) for 20 min at RT, washed with PBS and stained with 0.3 μg/mL DAPI for 10 min at RT. Finally, cells were washed with PBS and demineralized water, and embedded in Mowiol. For CD20 cluster analysis, PB B cells were fixed with 4% PFA prior to being adhered to PLL-coated coverslips. Imaging was performed using Zeiss LSM880 or Zeiss LSM900 confocal microscopes equipped with an Airyscan Detector and a 63× oil immersion 1.4 NA objective. Zeiss Zen software was used to control the microscope, to adjust spectral detection of DAPI emission and relevant fluorescent labels, and for subsequent Airyscan processing. Data analysis was conducted using Fiji image analysis software.

### Quantitative Determination of Cell Surface Antigens.

The total number of CD20 molecules at the cell surface of BJAB, Oci-Ly1, and PB B cells was quantified using an indirect immunofluorescence assay (QIFIKIT®, BioCytex) following the manufacturer’s instructions.

### FRAP of CD20, HLA-DR, and CD70.

BJAB (WT and CD70KO) and PB B cells were stained with antibodies against CD20 and HLA-DR, labeled with AF488, following the flow cytometry staining protocol. WT and CD20KO BJAB cells were transfected using the Neon transfection system (Thermo Fisher) with 2 µg eGFP-hCD70 (OHu23687C, Genscript). After the cells were stained or 24 h posttransfection, cells were washed and seeded in Willco dishes to attach for 15 min at 37 °C, prior to the experiment. FRAP was performed using a Leica TCS SP8 single molecule detection microscope equipped with a 60× water 1.2 NA objective (Leica) and an argon-ion laser set to bleach with 100% power at the 488 nm wavelength. The fluorescence intensity in the bleach zone as well as the whole cell and background was measured to correct for photobleaching and background signal, as previously described (68). Immobile and mobile fractions, as well as the recovery curve and speed of recovery (T-half), were calculated manually and confirmed using the easyFRAP web tool (70).

### TIRF Microscopy.

BJAB cells were stained with AF488 labeled CD20 antibodies following the flow cytometry protocol. Next, cells were washed and seeded on Wilco dishes coated with 2 M glycine for 15 min at 37 °C. Fluorescence imaging of CD20 was performed on the ventral side of cells using an Olympus IX71 inverted microscope working in TIRF geometry with a 150×, 1.45 NA oil objective. Fluorophore excitation was provided by a 488-nm solid-state laser Fluorescence was collected with the same objective and guided into an EM-CCD camera (Hamamatsu ImagEM) after suitable filtering. Movies were recorded at a frame rate of 10 Hz for a total of typically 1,000 frames. The sample temperature (34 to 36 °C) was maintained by a stage heater (Pecon) and an objective heater. Particles were tracked using the TrackMate plugin of Fiji (71), with a typical particle size of 0.35 microns squared.

### IP and Western Blot.

Dynabeads (Protein G, Invitrogen) were incubated with 3% BSA or IgG2a isotype control antibody, in PBS overnight at 4 °C, and subsequently washed with washing buffer [0.1% detergent (digitonin (Sigma-Aldrich) or Brij97 (Sigma-Aldrich)], 10 mM Tris-HCl (pH 7.5), 150 mM NaCl, 2 mM MgCl_2_, 2 mM CaCl_2_, protease (complete ULTRA, Roche), and phosphatase (PhosSTOP, Roche) inhibitors in MilliQ). BJAB cells were counted and collected, to get 10 × 10^6^ cells per sample. Cells were lysed in ice-cold lysis buffer for 45 min on ice, vortexed every 5 min. Lysis buffer contained 1% detergent (digitonin or Brij97), 10 mM Tris-HCl (pH 7.5), 150 mM NaCl, 2 mM MgCl_2_, 2 mM CaCl_2_, phosphatase, and protease inhibitors Na_3_VO_4_, NaF, and PMSF in MilliQ. Lysates were centrifuged at 6,000 rpm for 4 min at 4 °C, and supernatant was taken and precleared with BSA- and IgG2a-incubated Dynabeads for 1 h at 4 °C. Samples were centrifuged at 1,500 rpm for 20 s at 4 °C. Supernatant was split into two samples (small input sample was taken prior to splitting), and incubated for 1 h at 4 °C with 3 µg of anti-human CD20 antibody or the corresponding IgG2a isotype control antibody. Subsequently, samples were incubated for 90 min at 4 °C after addition of BSA-coated Dynabeads and washed using washing buffer using a magnetic Eppendorf-holder. Proteins were eluted using 2× sample buffer (made in house). Samples were boiled for 10 min at 70 °C and stored at −20 °C until they were used for western blot or MS. For western blot, reduced immunoprecipitated proteins were separated on a 12% sodium dodecyl sulfate (SDS) polyacrylamide gel and transferred to a PVDF membrane (GE Healthcare). The blot was blocked with Intercept-TBS (Li-Cor) for 1 h at RT and incubated with an anti-CD20 antibody at 4 °C overnight. CD20 was detected with a goat anti-mouse IRDye 800 secondary antibody after incubation for 1 h at RT. The blot was scanned using an Odyssey Infrared Imaging System (LI-COR Biosciences).

### CD20—CD70 Copurification.

Codon-optimized complementary DNA for expression in mammalian cells, encoding for CD70 and CD20 was obtained from GeneArt in a pMA-RQ and pJ201 plasmid, respectively. For expression in HEK293-E+ cells, full-length CD70 was subcloned into an expression vector containing a C-terminal triple repeat Strep II tag (U-Protein Express B.V.). CD20 was subcloned into an expression vector containing a C-terminal His_6_ tag (U-Protein Express B.V.). CD70 and CD20 were coexpressed in HEK293-E+ cells with a DNA ratio of 1:5 and harvested after 4 d. All subsequent steps were carried out at 4 °C. Cells were pelleted for 15 min at 1,000× g, washed in 1× PBS and subsequently lysed in Lysis buffer containing 50 mM K-PO_4_, pH 7.8, 200 mM KCl, 1% (m/v) n-dodecyl-ß-D-maltoside (DDM, Anatrace), protease inhibitor cocktail (Roche) for 2 h. The solubilized protein was separated from unbroken cells and cell debris by centrifugation for 5 min at 10,000× g, followed by an ultracentrifugation step for 45 min at 160,000× g. The supernatant was incubated with a Streptactin resin for 2 h. The resin was washed with 30 CV wash buffer (50 mM K-PO4, pH 7.8, 200 mM KCl, 0.025% DDM) and the protein eluted from the resin using wash buffer supplemented with 7 mM desthiobiotin (Sigma). The eluted fractions were pooled and concentrated using an Amicon Ultra Centrifugal Filter (Merk Millipore) with a cut-off size of 50 kDa. The concentrated protein was further purified using a Superose 6 10/300 increase GL gel filtration column (Cytiva) in SEC-buffer (20 mM HEPES/NaOH, pH 7.8, 150 mM NaCl, 0.025% DDM). The presence of both proteins was confirmed by western blot using the antibody dilutions of αHis 1:10,000 for CD20 and αStrep 1:1,000 for CD70.

### MS.

Prior to loading the coimmunoprecipitated proteins onto a Tricine-SDS-gel (10%), samples were resuspended in 10 µL of reducing loading buffer [12% SDS, 6% (v/v) β-mercaptoethanol, 30% (m/v) glycerol, 0.05% Coomassie blue G-250, 150 mM Tris/HCl, pH 7.0] and incubated for 15 min at 45 °C. The electrophoresis was carried out for 20 min at 50 V plus 10 min at 100 V (total migration distance ~0.5 cm). In parallel, a molecular mass marker (Precision plus protein standards, BioRad) was used to monitor the correct loading of proteins into the gel. Gel spots were cut, fixed, destained, and digested with pig trypsin (Promega) following the procedure described elsewhere (72). Resulting tryptic peptides were separated by liquid chromatography and analyzed by tandem MS in a Q-Exactive Orbitrap Mass Spectrometer equipped with an Easy nLC1000 instrument (Thermo Fisher) as also described by ref. 72. MS raw data files were analyzed using the MaxQuant software (v1.5.0.25) with the settings detailed elsewhere (73), except for the search against the Homo sapiens proteome retrieved from UniProt (01.01.2020) including known protein contaminants. Individual protein abundances were determined by label-free quantification and intensity-based absolute quantification (iBAQ) values.

### PLA.

BJAB cells were counted and collected at 100,000 cells per condition. Cells were adhered on PLL coated coverslips for 15 min at 37 °C in PBS, after which they were fixed with 4% PFA at RT for 20 min. Afterward, PLA was performed using Duolink In Situ detection reagents FarRed (Sigma-Aldrich) according to the manufacturer’s instructions. Cells were blocked with Duolink® blocking solution with 2% human serum, for 60 min at 37 °C. Then, cells were incubated with Duolink® Antibody Diluent with 2% human serum supplemented with primary antibodies against CD55, HLA-ABC, CD20, and CD70 for 30 min at 37 °C. After washing with Wash Buffer A, cells were stained with PLUS and MINUS PLA probes diluted 1:5 in Duolink® Antibody Diluent with 2% human serum for 1 h at 37 °C. Cells were washed again with Wash Buffer A before and after ligation was performed in 1× Duolink^®^ Ligation buffer for 30 min at 37 °C. Next, rolling circle amplification was performed using 1× FarRed amplification buffer for 100 min at 37 °C. After washing with Wash Buffer B, samples were stained with DAPI, washed, and embedded in Mowiol. Acquisition was done using Zeiss LSM880 using a 63× 1.4 NA oil immersion objective. Image analysis and PLA spot quantification was performed in Fiji.

### CD20 and CD70 Overexpression.

BJAB (WT, CD20KO, and CD70KO) and Oci-Ly1 (WT and CD20KO) cells were seeded 24 h prior to transfection at a density of 300,000 cells/mL. Equal amounts of cells were transiently transfected with hCD20-eGFP (OHu00965C, Genscript) or hCD70-eGFP (OHu23687C, Genscript), using the Neon transfection system (Thermo Fisher) according to the manufacturer’s instructions. 24 h after transfection, cells were collected for flow cytometry analysis or confocal microscopy and stained using specific antibodies against CD20 and CD70 as described above.

### Microcontact Printing.

BJAB cells were transiently transfected using the Neon transfection system (Thermo Fisher) according to the manufacturer’s instructions, and cells were imaged 24 h posttransfection with the indicated expression constructs. Poly(dimethylsiloxane) stamps containing a regular pattern of circular spots with a 5 μm diameter were made as described previously (46, 47, 68). Stamps were incubated for 1 h in PBS containing either CD27-Fc (Abcam) or IgG isotype control antibody, together with a donkey anti-rabbit IgG AF647 labeled secondary antibody to visualize the spots. After incubation, stamps were rinsed with demineralized water and dried using a nitrogen stream. The stamp was placed on a cleaned glass coverslip for 20 s and carefully removed. Next, transfected cells were plated onto the stamped surface and incubated for 30 min at 37 °C. Cells were fixed with 4% PFA for 20 min at RT. Samples were washed with PBS and demineralized water before being embedded in Mowiol. Cells were imaged using the DMI6000B inverted fluorescence microscope with the Leica DFC365 FX camera (HC PL APO 63×/1.40 OIL lens). The microscope was operated using Leica Application Suite Software and images were analyzed using Fiji image analysis software.

### Synapse Formation Using SEE or Cytomegalovirus Peptide.

BJAB cells (WT and CD20KO) and activated primary B cells (WT and CD20KO derived from healthy donors, or derived from a CD20-deficient patient) (50) were counted and collected at 100,000 (BJAB) or 50,000 (primary B cells) cells per condition. Cells were resuspended in PBS and plated in low-adherence 96-well plates. SEE (Toxin Technology) or Cytomegalovirus peptide (CMV, pp65-KYQEFFWDANDIYRI; HLA-DRB1*0101-binding peptide) was added to a final concentration of 2 μg/mL (SEE) or 1.5 μg/mL (CMV), and cells were then incubated for 20 min (BJAB) or 1 h (primary B cells) at 37 °C and washed with PBS. BJAB cells (WT or CD20KO, with or without SEE prestimulation) were cultured with Jurkat T cells. HLA-DR1+ activated primary B cells (WT or CD20KO, with or without CMV prestimulation) were cocultured with CMV-pp65-specific CD4^+^ T cells (51, 52), and activated primary B cells (WT or CD20KO, from healthy donors or derived from a CD20-deficient patient, with or without SEE prestimulation) were cultured with primary T cells from different donors.

To visualize synapse formation by microscopy, B and T cells were mixed to a 1:1 ratio and incubated for 2 h at 37 °C. Afterward, B cell:T cell complexes were carefully plated on PLL-coated (Sigma-Aldrich) coverslips for 15 min at RT and fixed using 4% PFA for 20 min at RT. Cells were stained for CD3, MHC II/HLA-DR, CD70, and CD20 as described for immunofluorescence microscopy. Cell complexes were imaged using a DMI6000B inverted fluorescence microscope equipped with a Leica DFC365 FX camera (HC PL APO 63×/1.40 OIL lens). The microscope was operated using Leica Application Suite Software and images were analyzed using Fiji image analysis software.

### T Cell Function Assays.

To study T cell activation and cytokine secretion upon synapse formation, cocultures of primary B and T cells were mixed on a 1:5 ratio, seeded onto round-bottom 96-well plates, and incubated for 1, 3, or 7 d. Prior to coculture, primary T cells were stained with CellTrace Violet (Thermo Fisher). Cells were collected at the specified time points and stained against CD25 for flow cytometry analysis. Supernatants were collected to determine IL-2 and IFN-γ levels using ELISA in accordance with the manufacturer’s instructions (Thermo Fisher).

### Image and Statistical Analysis.

Confocal image analyses were performed using Fiji image analysis software. Background on fluorescent images was removed by adjusting the brightness & contrast settings. Cells were randomly selected prior to image analysis, excluding blebbing cells. Manders’ overlap coefficient was calculated using Coloc2 or BIOP JACoP plug-in in Fiji, with bisection autothresholding. For the CD20 and CD70 cluster analysis, local maxima were found, followed by segmentation of maxima. Thresholding was applied [Otsu, 20% or autothreshold (CD20) or 30% (CD70)], and image was smoothed. Both segmented and thresholded images were combined using the image calculator. Holes arising were filled, using binary fill holes. Analyze particles plug-in in Fiji software was used to determine cluster size, number of clusters, and clustering intensity. Regions of interest (ROIs) were saved for reference. For PLA spot quantification, data were analyzed by first creating a threshold to distinguish the PLA spots from the background signal, then by analyzing particles with a size threshold and watershedding. To analyze CD70 recruitment in ISs formed by SEE in BJAB cells, top 1% of CD3 signal was taken to define synapses. ROIs were manually selected for interaction with BJAB cells (thus being interacting Jurkat and BJAB cells) by MHC II signal. Leftover ROIs were saved and overlaid with CD70 signal, to calculate number of synapses and CD70 signal within synapse. ROIs were manually moved to nonsynapse area of cell, five areas per images, to identify average CD70 signal out of synapse. Recruitment was defined as 150% or higher in-synapse CD70 signal compared to CD70 signal outside of synapse. Calculation was performed for MHC II using same ROIs. The number of B-T synapses was quantified by counting interactions between T cells (CD3+) and B cells (HLA-DR+) (WT or CD20KO). The total number of synapses was divided by the total number of B cells (WT or CD20KO) to determine the number of synapses per B cell.

All statistical analysis was performed using GraphPad Prism 8 software, and data are expressed as mean ± SEM or SD, as indicated in the figure legends. Statistical tests performed are specified in the figure legends. Statistical significance was set at *P* < 0.05.

## Supplementary Material

Appendix 01 (PDF)

Dataset S01 (XLSX)

## Data Availability

All study data are included in the article and/or supporting information.
