# Supplementary material for: CD70 recruitment to the immunological synapse is dependent on CD20 in B cells
Source: Proc Natl Acad Sci U S A. 2025 Apr 15;122(16):e2414002122. doi: 10.1073/pnas.2414002122 (PMC12037035; doi:10.1073/pnas.2414002122)
Supplement: Supplementary file 1 — Appendix 01 (PDF) [file pnas.2414002122.sapp.pdf]

CD70 recruitment to the immunological synapse is dependent on CD20 in B cells

Abbey B. Arp[a,1], Andrea Abel Gutierrez[a,1], Martin ter Beest[a], Guus A. Franken[a], Harry Warner[a], Andrea Rodgers Furones[a], Angelique N. Kenyon[a], Franziska Jäger[b], Alfredo Cabrera-Orefice[c], Kathrin Kläsener[d,e], Sjoerd van Deventer[a], Lenny Droesen[a], Vera Marie E. Dunlock[a,2], René Classens[a], Julian Staniek[e,f], Jannie Borst[g,h] , Michael Reth[d,e] , Ulrich Brandt[c], Piet Gros[b], Taco W. Kuijpers[i], Mirjam H. M. Heemskerk[j], Marta Rizzi[e,f,k,l], Laia Querol Cano[a,3], and Annemiek B. van Spriel[a,3,4], ✉ annemiek.vanspriel@radboudumc.nl

[a]Department of Medical BioSciences, Radboud Institute for Medical Innovation, Radboud University Medical Center, Nijmegen, 6525 GA, The Netherlands

[b]Department of Chemistry, Structural Biochemistry, Bijvoet Centre for Biomolecular Research, Faculty of Science, Utrecht University, Utrecht, 3584 CH, The Netherlands

[c]Radboud Institute for Medical Innovation, Radboud University Medical Center, Nijmegen, 6525 GA, The Netherlands

[d]Department of Molecular Immunology, Centre for Biological Signalling Studies and Centre for Integrative Biological Signalling Studies, Centers for Biological Signalling Studies, Biology III, Faculty of Biology, University of Freiburg, Freiburg, 79104, Germany

[e]Department of Rheumatology and Clinical Immunology, University Medical Center Freiburg, Faculty of Medicine, University of Freiburg, Freiburg, 79106, Germany

[f]Center for Chronic Immunodeficiency, University Medical Center Freiburg, Faculty of Medicine, University of Freiburg, Freiburg, 79106, Germany

[g]Department of Immunology, Leiden University Medical Center, Leiden, 2333 ZG, The Netherlands

[h]Oncode Institute, Leiden University Medical Center, Leiden, 2333 ZG, The Netherlands

[i]Department of Pediatric Immunology, Rheumatology and Infectious Diseases, Emma Children's Hospital, Amsterdam University Medical Center, Amsterdam, 1105 AZ, The Netherlands

[j]Department of Hematology, Leiden University Medical Center, Leiden, 2333 ZG, The Netherlands

[k]Division of Clinical and Experimental Immunology, Institute of Immunology, Center for Pathophysiology, Infectiology and Immunology, Medical University of Vienna, Vienna, 1090, Austria

[l]Centre for Integrative Biological Signalling Studies, University of Freiburg, Freiburg, 79104, Germany

[4]To whom correspondence may be addressed. Email: ✉ annemiek.vanspriel@radboudumc.nl.

[1] A.B.A. and A.A.G. contributed equally to this work.

[2]Present address: Department of Molecular Biology and Genetics, Johns Hopkins University School of Medicine, Baltimore, MMD 21205, USA.D.

[3]L.Q.C. and A.B.v.S. contributed equally to this work.

SFig. 1.

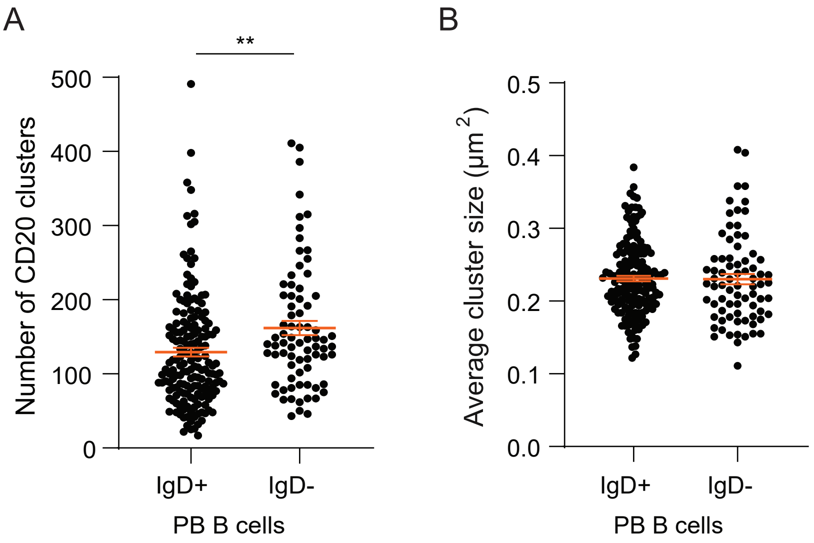

C

Cell type	Average Number of clusters in Imaged Area/ cell	Average Imaged Area/ cell	Average Diameter/ cell	Average Total Area/ cell	Average Total Number of clusters/ cell	Average Total Number of molecules/ cell	Average Number of molecules/ cluster
BJAB	123.41	111.33	14.50	600.86	666.05	13508.94	20.28
Oci-Ly1	66.16	49.15	14.22	641.71	863.71	35282.56	40.85
PB B cells	85.64	52.52	8.45	227	370.14	13924.05	37.62

SFig. 1. Additional CD20 cluster analysis. (**A**,**B**) Number of CD20 clusters (**A**) and the average CD20 cluster size (**B**) on IgD+ (naïve, unswitched memory) (n=173) and IgD- (switched memory) (n=76) B cells from peripheral blood of 5 different donors quantified in Airyscan confocal microscopy images. Statistical significance was assessed by Mann-Whitney U test. Mean +/- SEM is shown. (**C**) Table summarizing additional CD20 clustering parameters at the cell surface of BJAB, Oci-Ly1 and PB B cells, providing further information on the data shown in Fig. 1. Values represent the average from 3 independent experiments (BJAB and Oci-Ly1) and 5 donors (PB B cells).

SFig. 2.

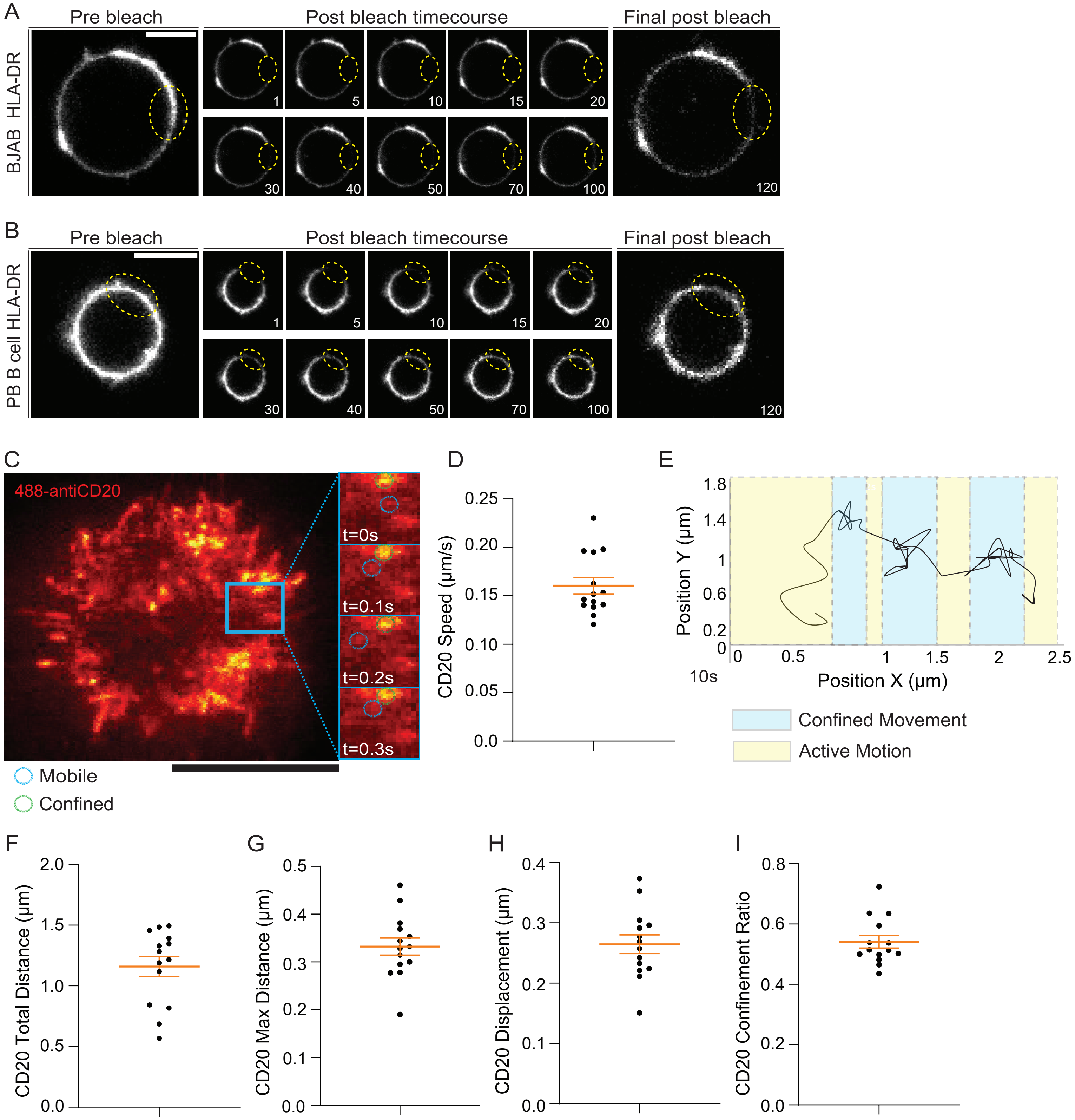

SFig. 2. CD20 particles are both mobile and confined. (**A**,**B**) Controls belonging to Fig. 2. Representative images of HLA-DR mobility by FRAP in BJAB (**A**) and PB B cells (**B**). Yellow circle indicating the bleached area and timing (seconds) as indicated in Fig. Scale bar: 5 μm. (**C**) Example TIRF image depicting a more and less mobile particle in BJAB cells. (**D**) Average Particle speed is just above 1.5 μm/s. (**E**) Example CD20 particle movement over 10 seconds. (**F**) Average Total Distance (full distance of a particle travelled for the movie's duration - 100 seconds) of CD20 particles. (**G**) Average Max Distance (the largest distance between the starting point and all subsequent points) travelled by a particle during one movie (100 seconds) (**H**) Average Particle Displacement (distance from starting position to final position) during one movie (100 seconds) (**I**) Average Particle Confinement Ratio of CD20 particles as defined by total distance divided by particle displacement. Average Data for 16 cells over 3 independent experiments. Scalebar: 10 μm. Mean +/- SEM is shown.

SFig. 3.

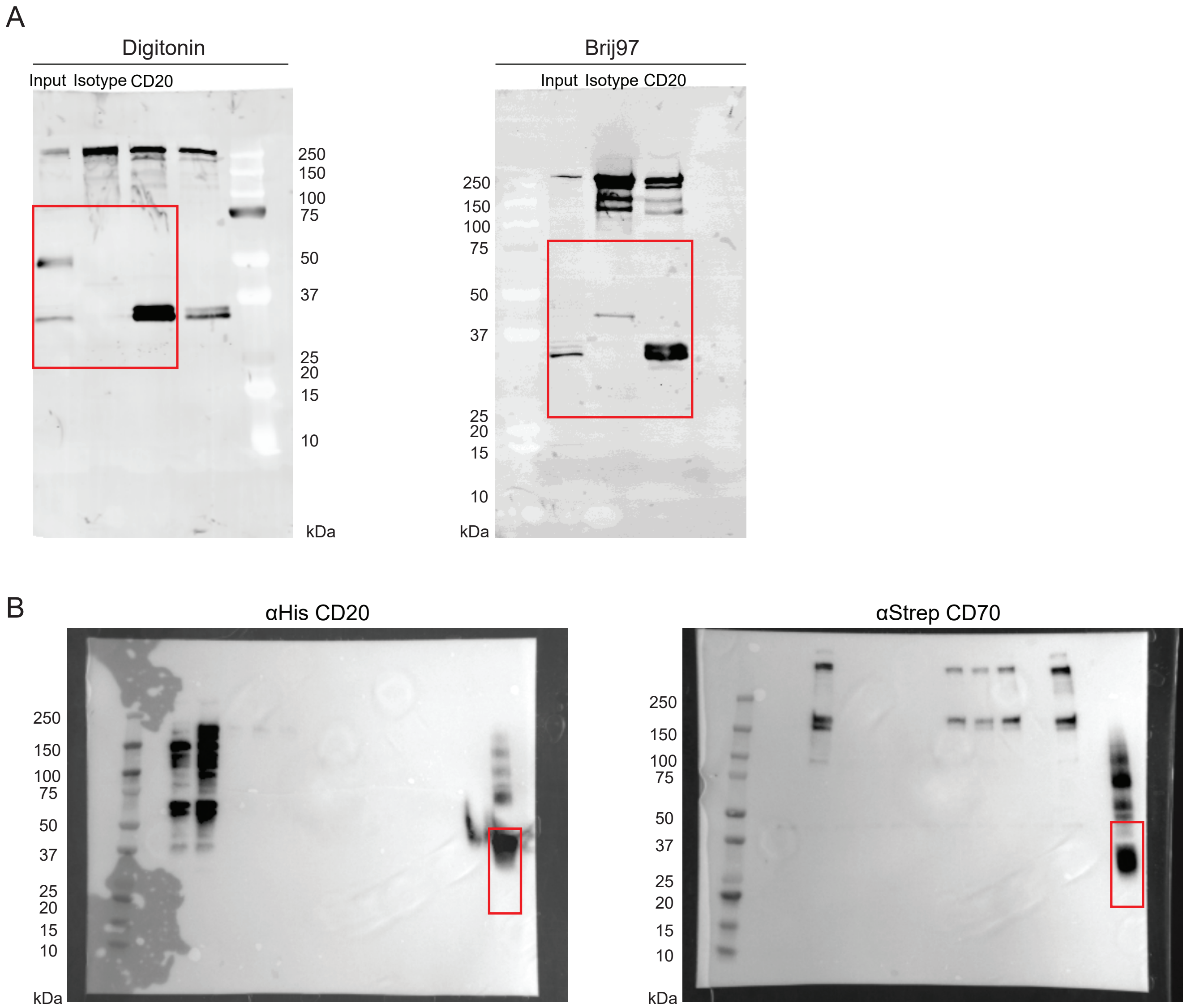

SFig. 3. Unprocessed original images of western blots. (**A**) Western blot corresponding to Fig. 3 A. (**B**) Western blot corresponding to Fig. 3 E.

SFig. 4.

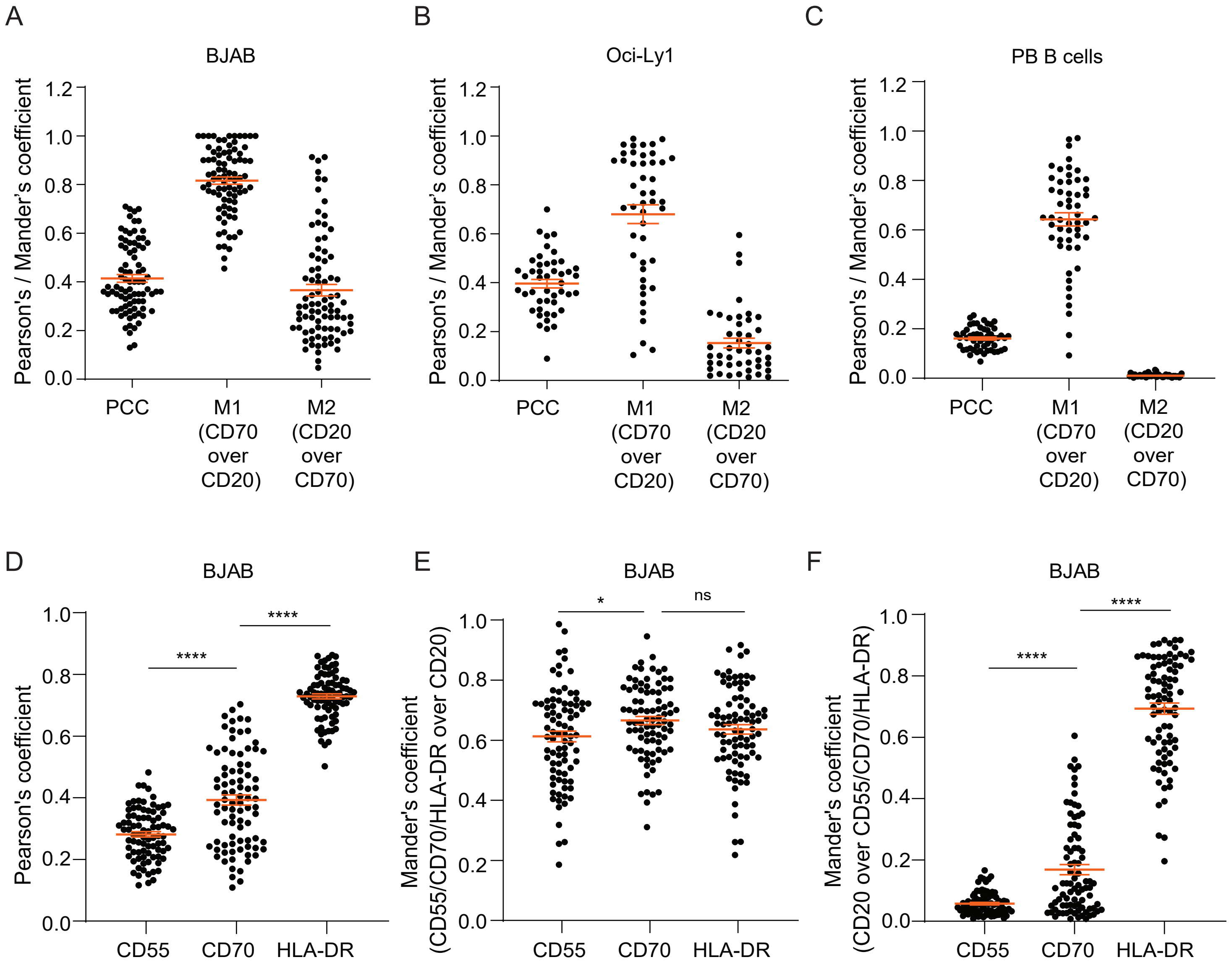

SFig. 4. CD20-CD70 colocalization analysis. (**A-C**) Quantification of CD20 and CD70 colocalization using Pearson's and Mander's overlap coefficients in BJAB (**A**), Oci-Ly1 (**B**) and PB B cells (**C**). Datapoints include 84 BJAB cells from 4 independent experiments, 46 Oci-Ly1 cells from 2 independent experiments, and 53 PB B cells derived from 3 independent donors. (**D-F**) Colocalization of CD20 with CD70, CD55 (negative control) and HLA-DR (positive control), analyzed by Pearson's coefficient (**D**), Mander's overlap coefficient for CD55/CD70/HLA-DR with CD20 (**E**), and Mander's overlap coefficient for CD20 with CD55/CD70/HLA-DR (**F**). Datapoints are 84 (CD55 and HLA-DR) or 85 (CD70) BJAB cells from 3 independent experiments. Statistical significance was assessed by one-way Anova with Tukey's multiple comparisons. Mean +/- SEM is shown.

SFig. 5.

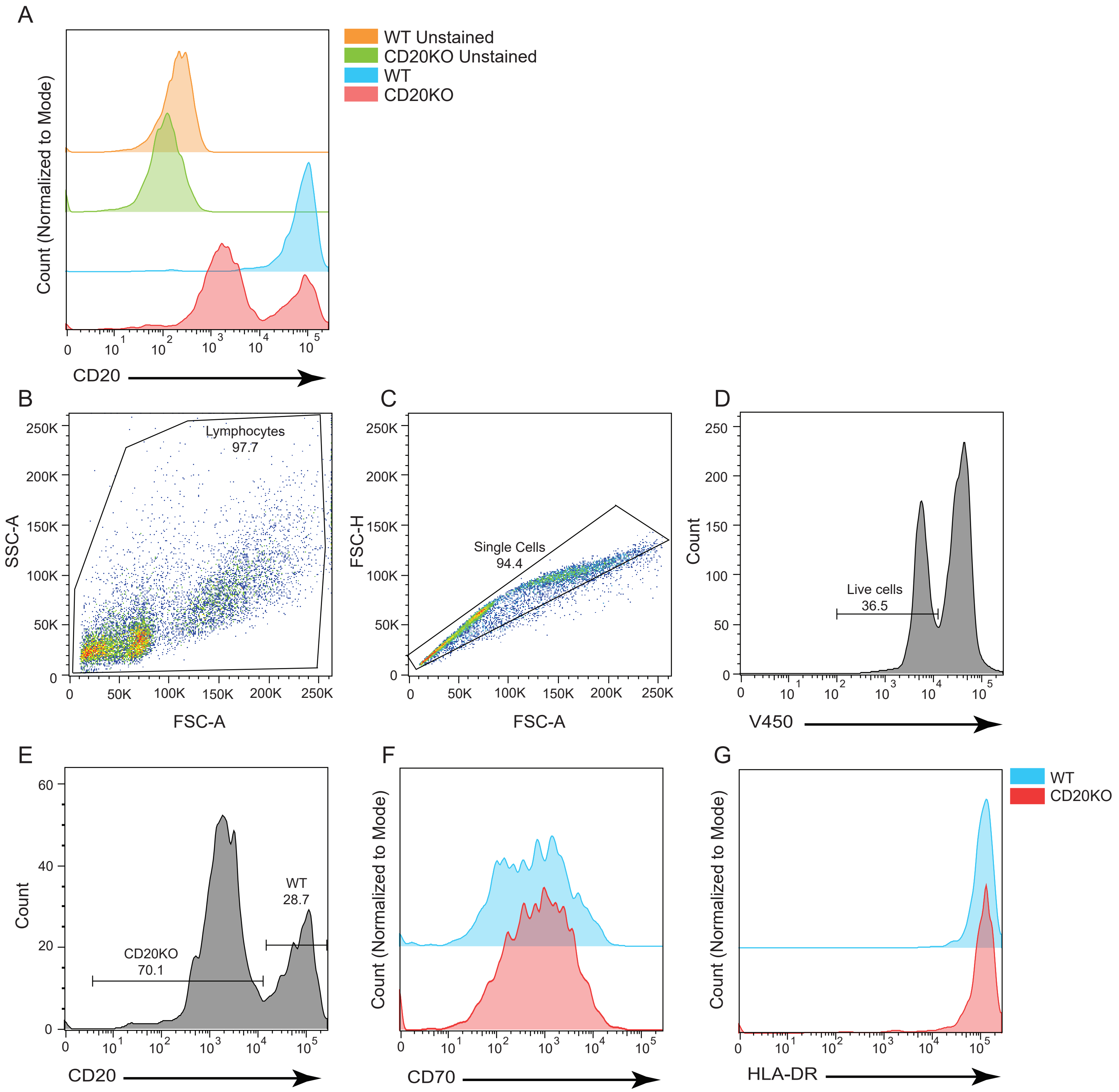

SFig. 5. Gating strategy and CD20KO characterization of primary B cells. (**A**) Representative histograms of CD20 surface expression in WT (unstained in orange, stained in blue) and CD20KO primary B cells (unstained in green, stained in red). (**B-E**) Gating strategy for CD20KO primary B cells, selecting the total population (**B**), single cell gating (**C**), exclusion of dead cells based on V450 negativity (**D**) and gating into WT or CD20KO based on the CD20 surface expression peaks (**E**). (**F-G**) CD70 (**F**) and HLA-DR (**G**) surface levels in WT (blue) and CD20KO (red) primary B cells.

SFig. 6.

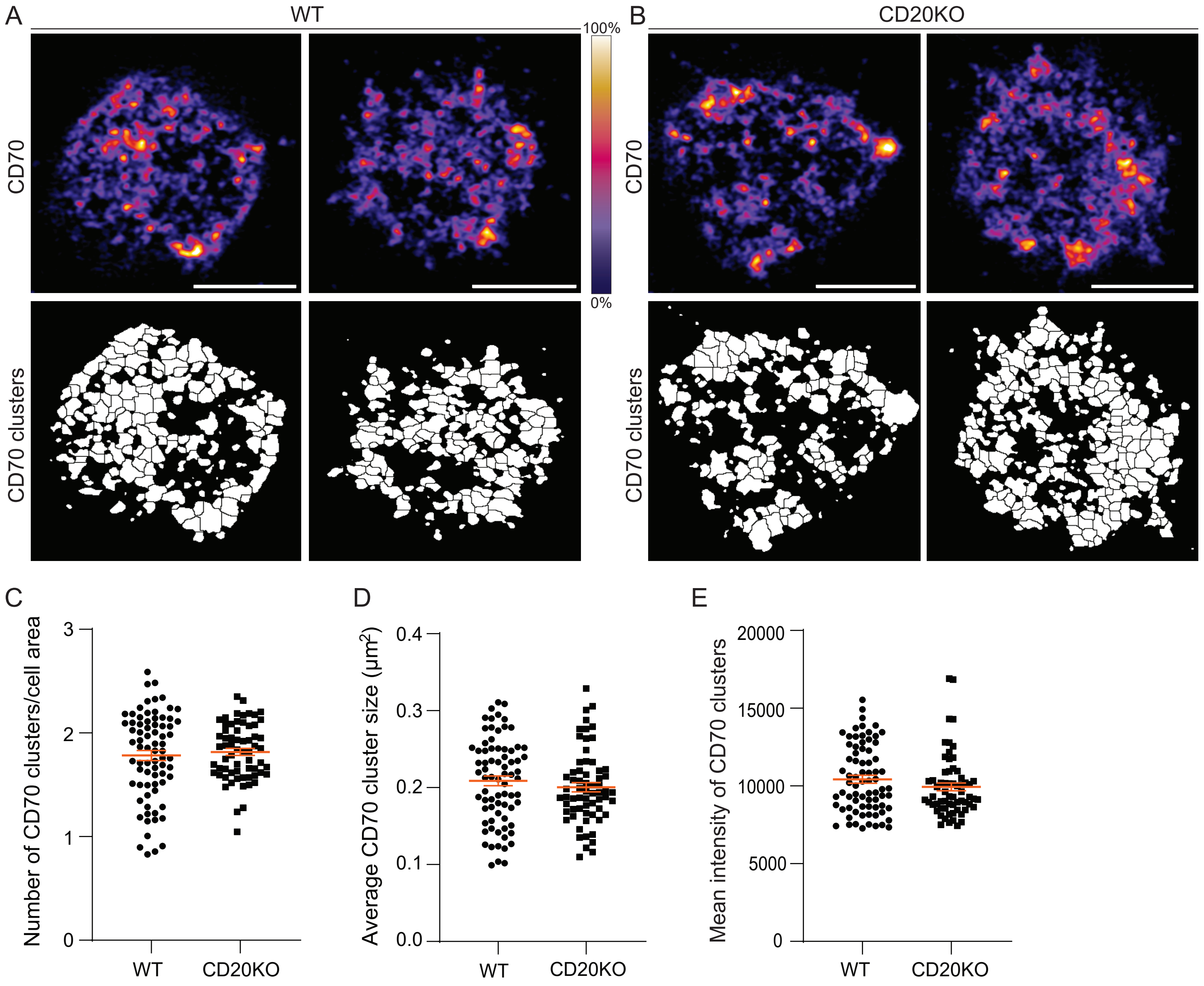

SFig. 6. CD20 loss does not affect CD70 clustering. (**A**,**B**) Representative confocal images of CD70 staining for WT (**A**) and CD20KO (**B**) BJAB cells. Scalebar: 5 μm. (**C**) Quantification of the number of CD70 clusters per cell area on bottom membrane of WT and CD20KO BJAB cells. Quantification of the size of CD70 clusters in μm2 (**D**) and their mean fluorescence intensity (**E**). Datapoints are 75 WT cells and 64 CD20KO cells derived from 2 independent experiments. Statistical significance was assessed by Mann-Whitney U test. Mean +/- SEM is shown.

SFig. 7.

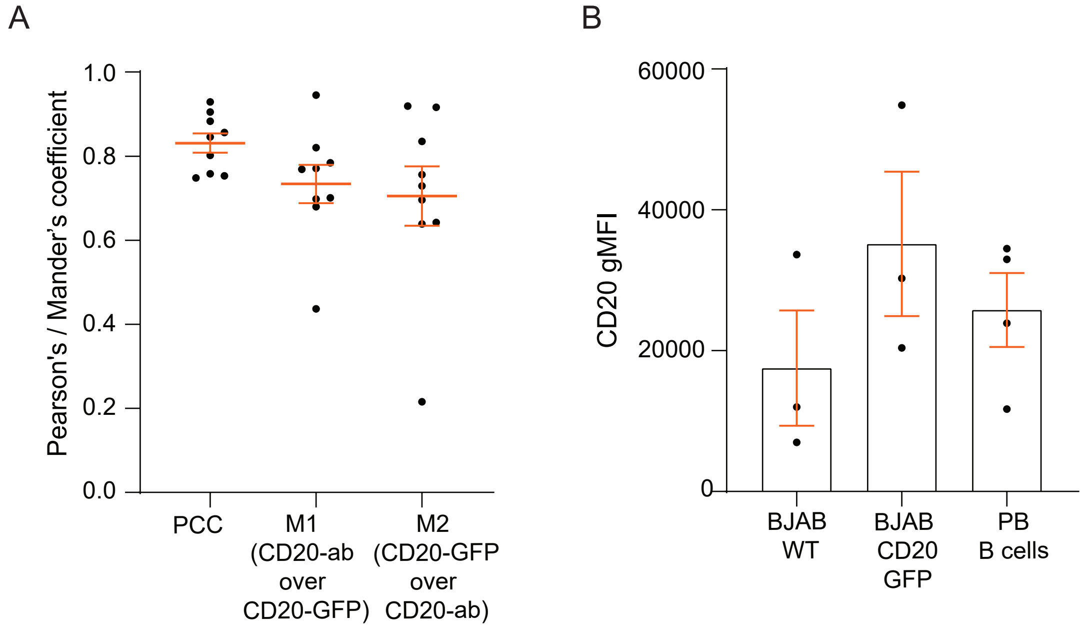

SFig. 7. CD20-GFP colocalizes with CD20 staining and slightly increases CD20 surface expression. (**A**) Quantification of CD20-GFP colocalization with CD20 staining in BJAB cells using Pearson's and Mander's overlap coefficients. Datapoints are 9 cells derived from 2 independent experiments. (**B**) CD20 surface expression levels in WT and CD20-GFP transfected BJAB cells, as well as in PB B cells, determined by flow cytometry. Datapoints are derived from 3 independent experiments (BJAB) and 4 donors (PB B cells). Statistical significance was assessed by one-way ANOVA. Mean +/- SEM is shown.

SFig. 8.

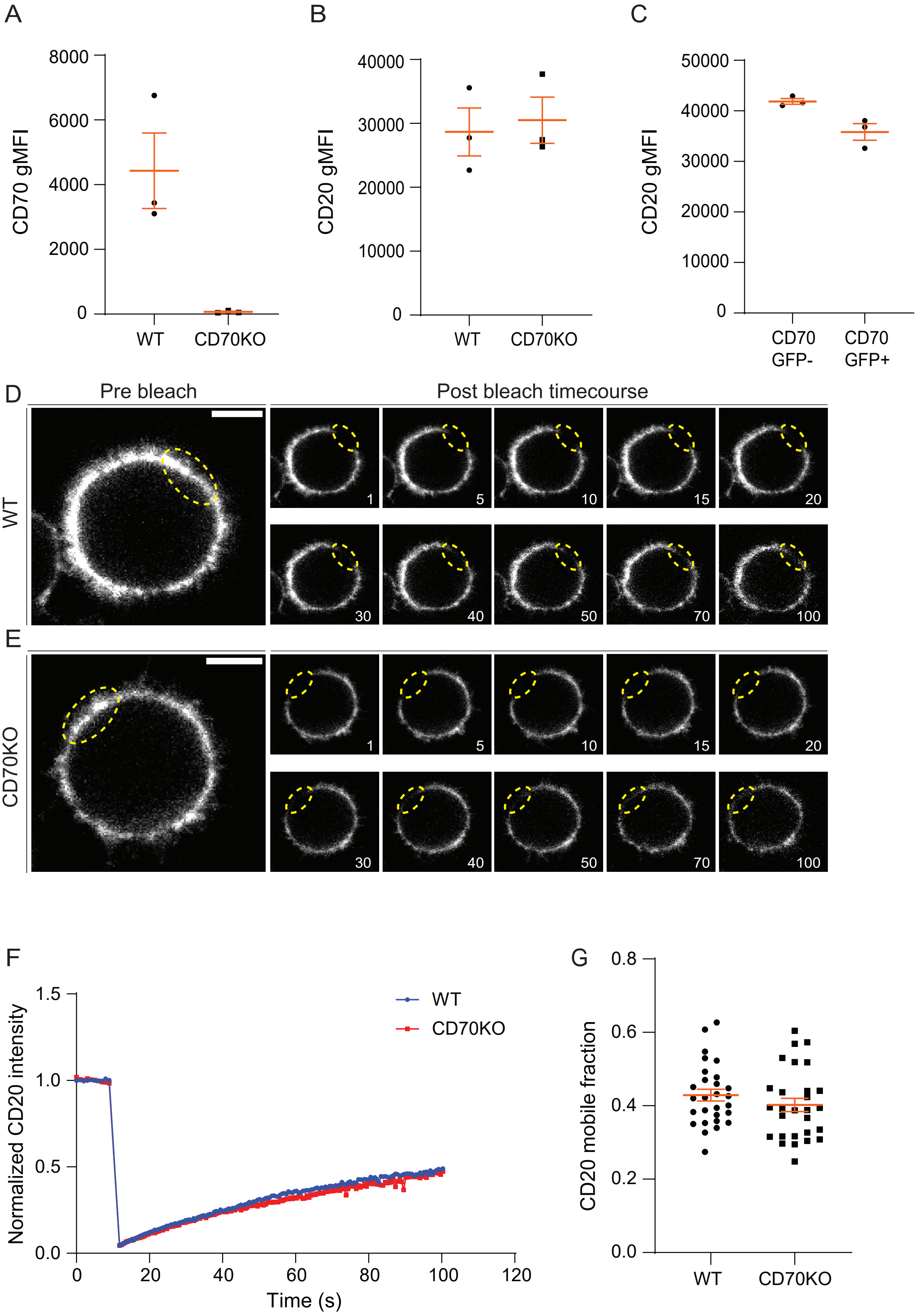

SFig. 8. Altered CD70 expression levels do not affect CD20 expression and mobility. (**A,B**) Quantification of CD70 (**A**) and CD20 (**B**) MFI values in WT and CD70KO BJAB cells, normalized to isotype. Datapoints derived from 3 individual experiments. (**C**) Quantification of CD70 MFI value in BJAB cells transfected with CD70-GFP, gated for GFP+ or GFP-, normalized to isotype. Datapoints derived from 3 individual experiments. (**D,E**) Representative confocal images of CD20 FRAP time course for WT (**D**) and CD70KO (**E**) BJAB cells. CD20 signal and distribution pre bleach (left), followed by several images during post bleach imaging (right). Yellow circle indicating the bleached area and timing (seconds) as indicated in Fig. Scalebar: 5 μm. (**F**) Mean recovery curve of CD20 signal in WT (blue) and CD70KO (red) BJAB cells, normalized to prebleach intensity. (**G**) Quantification of CD20 mobile fraction in WT and CD70KO BJAB cells. Datapoints are 34 cells analyzed for both conditions, derived from 3 individual experiments. Statistical significance was assessed by Mann-Whitney U test (**F**). Mean +/- SEM is shown.

SFig. 9.

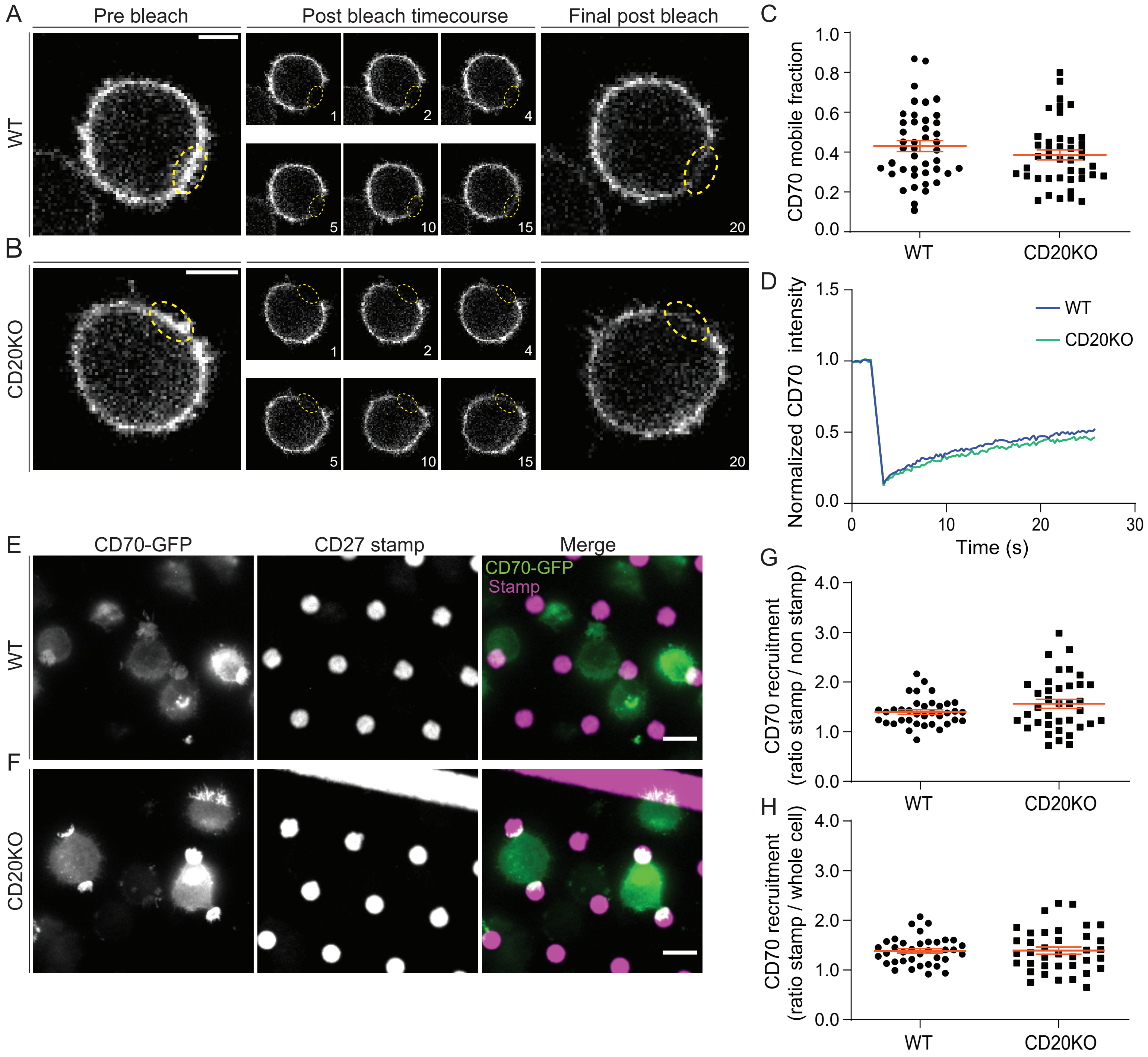

SFig. 9. CD20 knock-out does not affect CD70 mobility or recruitment towards CD27 in steady state. (**A, B**) Representative confocal images of FRAP time course for WT (**A**) and CD20KO (**B**) BJAB cells. Shown is CD70 signal and distribution pre bleach (left), followed by several images during post bleach imaging (middle), and final post bleach image (right). Yellow circle indicating the bleached area and timing (seconds) as indicated in Fig. Scale bar: 5 µm. (**C**) Quantification of CD70 mobile fraction in WT and CD20KO BJAB cells. (**D**) Mean recovery curve of CD70 signal in WT (blue) and CD20KO (green) BJAB cells, normalized to prebleach intensity. More than 40 cells were analyzed for both conditions derived from 3 independent experiments. (**E, F**) WT (**E**) and CD20KO (**F**) BJAB cells expressing CD70-GFP (green, left) were seeded on CD27 stamps (magenta, middle) for 30 min and imaged using epifluorescence. Merge shown on right. Scalebar: 10 µm. (**G**) Quantification of ratio between CD70-GFP intensity on the CD27 stamp and outside stamp for WT and CD20KO BJAB cells. (**H**) Quantification of ratio between CD70-GFP intensity on the CD27 stamp and whole cell for WT and CD20KO BJAB cells. Datapoints are 37 cells analyzed for both conditions derived from 2 independent experiments. Experiments were performed 4 times, yielding similar results. Statistical significance was assessed by Mann-Whitney U test (**C**), unpaired t-test (**G, H**), mean ± SEM is shown.

SFig. 10.

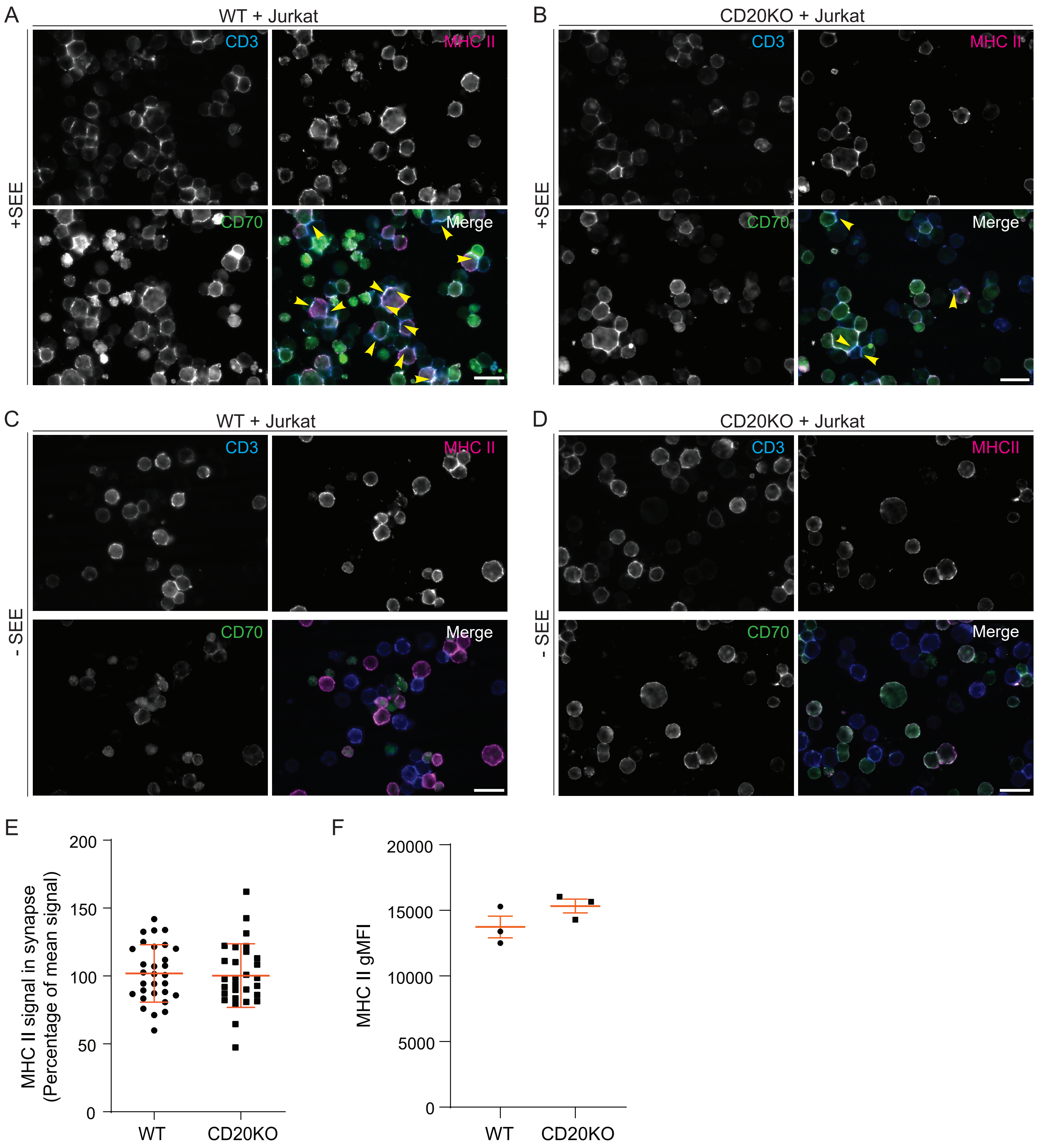

SFig. 10. Controls belonging to Fig. 5. (**A-D**) Representative epifluorescence images of co-cultures with Jurkat and WT (**A,C**) or CD20KO (**B,D**) BJAB cells with (**A,B**) and without (**C,D**) staphylococcal enterotoxin E (SEE). Shown is CD3 (blue, top left), MHC II (magenta, top right), CD70 (green, bottom left) and merge (bottom right). Yellow arrows indicate immune synapses. Scalebar: 25 µm. (**E**) Quantification of MHC II intensity in CD3-enriched areas as a percentage of mean MHC II intensity in co-cultures between Jurkat T cells and WT or CD20KO BJAB cells. Datapoints are derived from 3 individual experiments. 10 images per condition were analyzed ranging between 300-400 synapses per condition. (**F**) Quantification of MHC II expression by flow cytometry (MFI) in WT and CD20KO BJAB cells. Significance was assessed by unpaired t-test, mean ± SEM is shown.

SFig. 11.

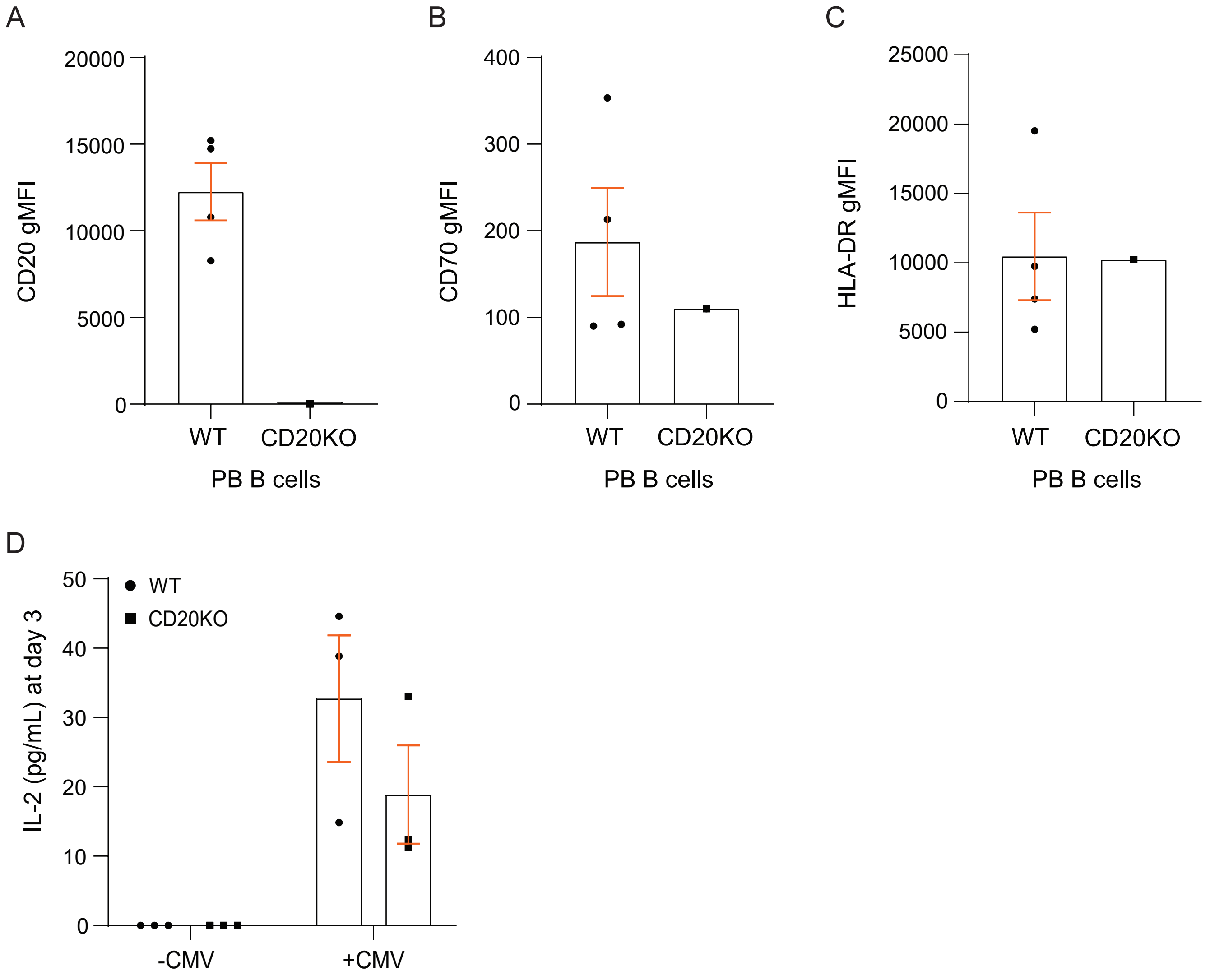

SFig. 11. Additional data for Fig. 6. (**A-C**) Surface expression of CD20 (**A**), CD70 (**B**), and HLA-DR (**C**) in activated primary B cells from a CD20-deficient patient (CD20KO) and 4 healthy donors (WT), determined by flow cytometry. (**D**) IL-2 secretion by anti-CMV specific CD4+ T cells 3 days after synapse formation with WT or CD20KO primary B cells, determined by ELISA. Datapoints represent 3 independent donors. Statistical significance was assessed by two-way ANOVA with Sidak's multiple comparisons test. Mean +/- SEM is shown.

SFig. 12.

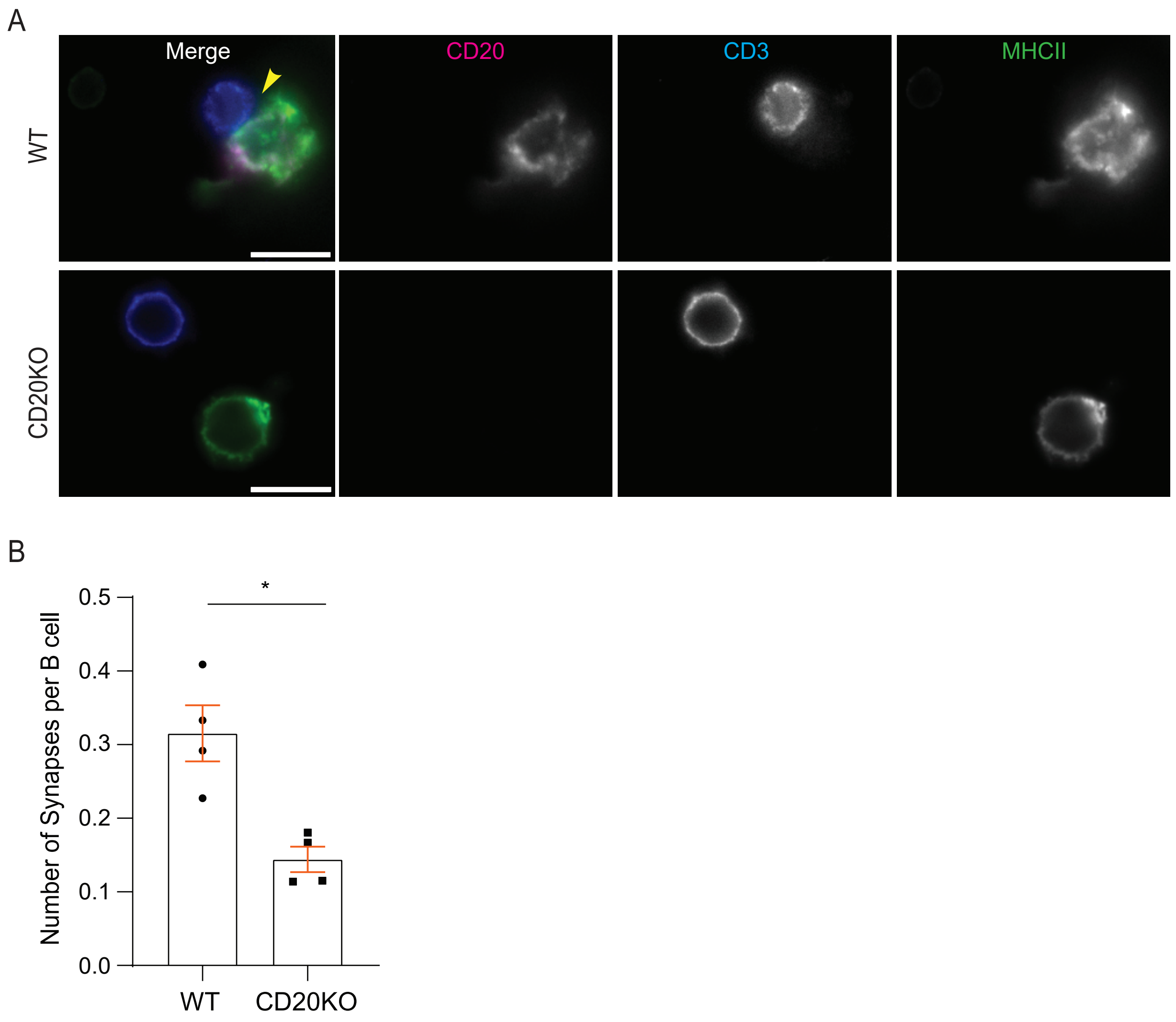

SFig. 12. CD20 loss in primary B cells impairs synapse formation in mixed lymphocyte reactions. (**A**) Representative epifluorescence images of co-cultures of primary allogenic T cells with WT (top) or CD20KO (bottom) activated primary B cells. Shown are merged images (left), CD20 (magenta, middle left), CD3 (blue, middle right) and HLA-DR (green, right). Yellow arrows indicate immune synapses. Scalebar: 10 µm. (**B**) Quantification of the number of synapses per B cell in co-cultures of primary B and T cells from different donors. Datapoints represent 4 donors. 63 images were analyzed. Statistical significance was assesed by paired t-test. Mean ± SEM is shown.

STable.

	Antibody	Source	Identifier	Use
Primary Antibodies	anti-human CD20	Abcam	Clone: L26<br>Cat#: AB9475	IP: 3 µg<br>WB: 1:200
	anti-human CD20	Biolegend	Clone: 2H7<br>Cat#: 302302	FC: 20 µg/m
	anti-human CD20	Invitrogen	Clone: 001<br>Cat#: MA536001	PLA: 10 µg/ml
	anti-human CD20-APC	eBioscience	Clone: 2H7<br>Cat#: 17-0209-42	FC: 1:5
	anti-human CD20-BV421	BD Biosciences	Clone: 2H7<br>Cat#: 404-0209-42	FC: 1:10
	anti-human CD20-AF647	Biolegend	Clone: 2H7<br>Cat#: 302318	FC/IF: 1:3.33
	anti-human CD20-AF488	Biolegend	Clone: 2H7<br>Cat#: 302316	FRAP/FC/TIRF: 10 µg/ml
	anti-human CD20-FITC	BD Biosciences	Clone: L27<br>Cat#: 340673	FRAP: 10 µg/ml
	anti-human CD70	Abcam	Clone: BU69<br>Cat#: AB77868	FC: 10 µg/ml<br>IF: 20 µg/ml<br>PLA: 10 µg/ml
	anti-human CD70-AF647	Biolegend	Clone: 113-16<br>Cat#: 355116	FC: 10 µg/ml<br>IF: 20 µg/ml IF
	anti-human HLA-DR, DP,DQ -FITC	BD Biosciences	Clone: Tu39<br>Cat#: 555558	IF: 1:50
	anti-human CD3-BV421	Biolegend	Clone: SK7<br>Cat#: 344834	IF: 1:50
	anti-human IgD-AF488	Biolegend	Clone: IA6-2<br>Cat#: 348216	IF: 10 µg/ml
	anti-human CD55	Invitrogen	Clone: BRIC-216<br>Cat#: MA191161	PLA: 10 µg/ml
	anti-human HLA-ABC	BD Biosciences	Clone: G46-2.6<br>Cat#: 555551	PLA: 10 µg/ml
	anti-human HLA-DR-BV510	Biolegend	Clone: L243<br>Cat#: 307646	FC: 1:20
	anti-human HLA-DR-AF488	Biolegend	Clone: L243<br>Cat#: 307656	FRAP/FC/IF: 10 µg/ml
	anti-human CD25-PECy7	Biolegend	Clone: BC96<br>Cat#: 302612	FC: 1:50
	Anti-His	Sigma Aldrich	Cat#: SAB1305538	CP: 1:10 000
	Anti- His	Qiagen	Cat#: 34650	CP: 1: 10 000
	Anti-Strep	IBA	Cat#: 2-1507-001	CP: 1: 1 000
Isotype Control Antibodies	mouse-IgG2a,k	Biolegend	Clone: MOPC-173<br>Cat#: 400202	IP: 3 µg
	mouse-IgG2a,k-FITC	Biolegend	Clone: MOPC-173<br>Cat#: 400208	IF: 1:50
	mouse-IgG2b,k-APC	eBioscience	Clone: eBMG2b<br>Cat#: 17-4732-42	IF: 1:50
	mouse-IgG2b-AF647	Biolegend	Clone: MPC-11<br>Cat#: 400330	FC/IF: 10 µg/ml
	mouse-IgG2b,k-AF488	Biolegend	Clone: MPC-11<br>Cat#: 400329	FC: 10 µg/ml
	mouse-IgG1,k-AF647	eBioscience	Clone: p3.6.2.8.1<br>Cat#: 51-4714-81	FC: 10 µg/ml<br>IF: 20 µg/ml IF
	mouse-IgG2a,k-BV510	Biolegend	Clone: MOPC-173<br>Cat#: 400267	FC: 1:20
	mouse-IgG1,k	Biolegend	Clone: MOPC-21<br>Cat#: 400102	FC: 10 µg/ml<br>IF: 20 µg/ml IF
	mouse-IgG1-FITC	BD Biosciences	Clone: X40<br>Cat#: 340755	FC: 10 µg/ml
	Rabbit IgG	Cell Signaling Technology	Clone: DA1E<br>Cat#: 3900S	MCP: 100 µg/ml
Secondary Antibodies	goat-anti-mouse IgG1-AF568	Invitrogen	Cat#: A21124	FC/IF: 1:400
	goat-anti-mouse IgG1-AF488	Invitrogen	Cat#: A21121	FC/IF: 1:400
	goat-anti-mouse IgG1-AF647	Invitrogen	Cat#: A21240	FC/IF: 1:400
	goat-anti-mouse IgG2a-AF488	Invitrogen	Cat#: A21241	FC/IF: 1:400
	donkey anti-rabbit IgG (H&L)-AF647	Invitrogen	Cat#: A31573	MCP: 1:400
	goat-anti-mouse IRDye800	Li-Cor	Cat#: 926-32210	WB: 1:5 000

STable. IP: Immunoprecipitation/ WB: Western Blot/ PLA: Proximity Ligation Assay/ FC: Flow Cytometry/ IF: Immune Fluorescence/ FRAP: Fluorescence Recovery After Photobleaching/ TIRF: Total Internal Reflection Fluorescence/ CP: Co-Purification/ MCP: Micro-contact Printing
